# Proteomic analysis of secreted proteins derived from amniotic fluid stem cells

**DOI:** 10.1007/s00441-025-03984-0

**Published:** 2025-06-07

**Authors:** Tatsanee Phermthai, Puttachart Chuaynarong, Suparat Wichitwiengrat, Kamonpat Phermthai, Sittiruk Roytrakul, Thanuch Chitthira, Sasiprapa Thongbopit

**Affiliations:** 1 Stem Cell Bank Unit, Research Department, Faculty of Medicine Siriraj Hospital, Mahidol University, 2 Prannok Road, Siriraj, Bangkoknoi, Bangkok, 10700 Thailand; 2 Suankularb Wittayalai School, Wang Burapha Phirom, Phra Nakhon, Bangkok, Thailand; 3 National Center for Genetic Engineering and Biotechnology (BIOTEC), National Science and Technology Development Agency, Pathum Thani, Khlong Luang Thailand

**Keywords:** Extracellular vesicle, Neuron, Regenerative medicine, Stem cell, Therapeutics

## Abstract

Mesenchymal stem cells (MSCs) show promising therapeutic effects due to the proteins they secrete. However, MSCs from different sources exhibit only 60% similarity of proteins they secrete, suggesting that unique proteins may offer distinct therapeutic properties based on their origin. Amniotic fluid-derived MSCs (AFSCs) are promising for treating degenerative diseases and are unique in providing sufficient cells for fetal therapies. Nevertheless, their proteomic profiles remain largely undefined. This study investigated the proteomic profiles of bioactive molecules secreted by AFSCs (AFSC-se) using liquid chromatography and mass spectrometry, along with bioinformatics tools for protein function analysis. We identified over 2000 proteins in the AFSC-se that are involved in various mechanisms supporting organ development and function. The top three proteins identified were associated with organelle fusion, forebrain morphogenesis, and response to parathyroid hormone. Our findings indicate that AFSC-se has the ability to inhibit inflammation and apoptosis, which corresponds to 7.8% of the identified proteins involved in pathways related to these therapeutic effects. Furthermore, we discovered that 20% of identified proteins are associated with brain functions including synaptogenesis, neurogenesis, and neuroprotection. In conclusion, the proteomic profile of AFSC-se indicates its potential therapeutic effects. The significant presence of neuro-related proteins in AFSC-se suggests that AFSC-se may be a promising candidate for treating neurological diseases. Our work addresses existing knowledge gaps in this field.

**Supplementary Information:**

The online version contains supplementary material available at 10.1007/s00441-025-03984-0.

## Introduction

Mesenchymal stem cells (MSCs) have great potential for treating degenerative diseases. The therapeutic effects of MSCs are primarily anti-inflammatory, antiapoptotic, promoting cell proliferation, and pro-angiogenic (Corcelli et al. 2018; Otani et al. 2019; Sato et al. 2020; Zou et al. 2023). MSCs facilitate the healing effect through their secretory bioactive molecules (Han et al. 2022). The secreted molecules, known as the secretome, consist of two main components that provide therapeutic effects of MSCs: extracellular vesicles (EVs) and soluble factors. The EVs contain paracrine factors, such as miRNA, transcriptional factors, and DNA, which help cells communicate with each other and influence biological processes in recipient cells. The soluble factors in the secretome include cytokines; chemokines; growth factors; anti-inflammatory mediators like IL-1ra, IL-10, and IL-13; and angiogenic factors like angiopoietin-1 and endostatin (D'Arrigo et al. 2019; Katifelis et al. 2022; Kou et al. 2022; Marassi et al. 2024). The secretory proteins released by MSCs play a crucial role in treating various diseases, including wounds, respiratory dysfunction, and inflammation (Goo et al. 2024; Han et al. 2022). However, bioactive molecules present in MSCs from different sources could be different. In a study by Angulski et al. (2017), it was found that secretory molecules derived from human umbilical cord blood stem cells and bone marrow stem cells showed only 60% similarity in all secreted proteins. These proteins are mainly associated with the functions of cell growth and antioxidative stress (Angulski et al. 2017).

Amniotic fluid-derived mesenchymal stem cells (AFSCs) are a specific type of stem cell derived from fetus during the 16 to 24 weeks of gestation, a dynamic period for the growth and development of fetal organs. Their unique origin gives AFSCs superior growth and proliferation capabilities compared to other types of MSCs. Importantly, AFSCs are the only MSC source that can be collected during pregnancy, providing an adequate supply of cells for preterm infants and fetal therapies. This allows for the possibility of autotransplantation preparations before delivery. (Abe et al. 2023). Research by Antounians et al. (2024) demonstrated that EVs derived from AFSCs, when injected into the amniotic cavity, can reverse symptoms of pulmonary hypoplasia in a congenital diaphragmatic hernia model. A study by Figueira et al. (2024) showed an improvement in lung function in neonatal rats following intra-amniotic injections of AFSCs biological fluid containing EVs. Manni et al. (2024) presented findings indicating that bioactive molecules released by AFSCs play a role in modulating autoimmune responses in a mouse model of multiple sclerosis. Additionally, secretory proteins derived from AFSCs have been effectively used in treating various medical conditions, such as cardiac impairment (Balbi et al. 2019), necrotizing enterocolitis (Ning et al. 2023), pulmonary hypoplasia (Antounians et al. 2024), and osteoarthritis (Zavatti et al. 2020). These findings suggest a promising potential for developing a future non-cellular stem cell therapy based on AFSC-se.

While the bioactive molecules secreted by AFSCs show promise in regenerative medicine, there is currently limited information regarding their protein profiles. Understanding the proteomic composition of these secretory bioactive molecules is a crucial step toward gaining insights for future therapeutic applications. Therefore, this study aims to investigate the proteomic composition and functional roles of AFSC-secreted molecules via bioinformatic analysis. This research will help address existing knowledge gaps in the field to support the future therapeutic use of AFSCs.

## Materials and methods

### AFSC providing

AFSC lines were derived from human amniotic fluid collected through genetic amniocentesis, performed for routine fetal genetic determination at 16–17 weeks of gestation in the Department of Obstetrics and Gynecology, Faculty of Medicine Siriraj Hospital. Informed consent was obtained from all subjects and/or their legal guardians. The study adhered to the 1964 Declaration of Helsinki. The AFSC derivation and collection of AFSC conditioned medium received approval from the Ethics Committee of Siriraj Hospital, Mahidol University (Si 269/2022). AFSC lines (*n* = 4) at passage 7 were expanded in minimum essential medium α (α-MEM; Gibco, Invitrogen, CA) supplemented with 20% amniomax-II complete medium (Gibco), 15% embryonic stem cell-qualified fetal bovine serum (ES-FBS; Sigma-Aldrich, Merck, Darmstadt, Germany), 1% L-glutamine (Gibco), and 1% penicillin–streptomycin (Gibco) at 37 °C and 5% CO_2_ until they reached 70–80% confluence. The characteristics of MSCs were confirmed by cell surface markers using flow cytometry and tri-lineage differentiation.

### Isolation and identification of AFSC-se

The AFSCs were cultured for two days in 10 ml of α-MEM supplemented with 10% exosome-free FBS (Gibco). After incubation, the culture medium was collected and centrifuged at 300 g for 5 min, followed by a second centrifugation at 3000 g for 5 min at 4 °C. To obtain the secretory proteins from the AFSCs for our study, we concentrated the proteins in the supernatant using centrifugal filter units with a 10 kDa cutoff (Pall Corporation, Cytiva, NY). This was done with centrifugation at 5000 g for 15 min at 4 °C. The isolated secretome was then collected and stored at − 80 °C for further experiments. To analyze the concentration and size distribution of particles in the AFSC secretome, we employed nanoparticle tracking analysis (NTA) in a NanoSight NS300 (Malvern Panalytical, Malvern, UK). The data were processed using NTA software version 3.4.4 and presented as mean ± standard deviation (SD). The total protein concentration in the AFSC-se was measured using the Bradford assay. For analysis of EV markers, the sample underwent Western blot analysis to detect the presence of CD9, CD63, and Annexin A1. A 20 µg of AFSC-se protein was separated through 12% SDS-PAGE. Subsequently, the proteins were transferred to a PVDF membrane. The membrane was blocked and incubated with mouse anti-beta actin (1:1000, ab8226, Abcam), rabbit anti-CD9 (1:1000, ab263019, Abcam), rabbit anti-CD63 (1:1000, ab134045, Abcam), and rabbit anti-annexin A1 (1:1000, #32934, Cell Signaling) overnight. The membrane was then hybridized with secondary HRP-conjugated anti-mouse (1:20,000, ab97046, Abcam) or HRP-conjugated anti-rabbit (1:10,000, ab6721, Abcam). The blot was visualized by the ImageQuant LAS 4010 system (GE Healthcare Life Sciences).

### Anti-inflammation assay

Human THP-1 monocytes were placed into 96-well plates at an initial density of 100,000 cells/well and cultured in Roswell Park Memorial Institute medium (RPMI 1640; Gibco) supplemented with 10% FBS (Gibco) and 1% penicillin–streptomycin (Gibco). They were then differentiated into M0 macrophages by incubation with 5 ng/ml phorbol 12-myristate 13-acetate (PMA; Sigma) for 24 h, followed by 72 h of resting in a 10% RPMI medium. The M0 were polarized into M1 by exposure to 10 ng/ml LPS (Sigma) and 20 ng/ml IFN-γ (Sigma). After 18 h of polarization, M1 was treated with 1 × 10^9^ particles/ml AFSC-se for 6 h. Subsequently, the medium was collected to analyze the release of IL-1β from macrophages using Lumit® IL-1β human immunoassay (Promega, WI) according to the manufacturer’s protocols.

### Cell apoptosis assay

Dermal fibroblast cells were initially seeded into a 6-well plate at a density of 60,000 cells per well in 10% DMEM and then incubated for 24 h. Following this, the cells were exposed to 400 µM H_2_O_2_ for 24 h and then treated with 5 × 10^9^ particles per well of AFSC-se for 48 h of incubation. After the incubation, the cells were harvested and stained using an Annexin V-FITC/PI flow cytometry assay kit (Invitrogen, Gibco) according to the manufacturer's instructions. In brief, cells were centrifuged at 300 g for 5 min. The cell pellet was then resuspended in 1X Annexin-binding buffer and stained with Annexin V-FITC reagent and PI reagent. After incubating in the dark for 15 min at room temperature, the cells were added with annexin-binding buffer before being analyzed using a FACSCalibur flow cytometer (Becton Dickinson, San Jose, CA). The apoptotic rate (AR) was calculated using the formula AR = (NSC − NVC)/NSC, where NSC is the number of seeded cells and NVC is the number of annexin V-negative cells.

### LC–MS/MS analysis

The sample was purified using a Clean-up kit (GE Healthcare, USA) to obtain a protein pellet to prepare the AFSC-se sample for LC–MS. The pellet was then lysed in urea lysis buffer solution (8 M urea in 0.1 M Tris -HCl), and the protein concentration was measured by Bradford’s method (Bio-Rad protein assay, Bio-Rad Laboratory, CA). A 30 µg protein sample was reduced in a reduction buffer (100 mM dithiothreitol in 100 mM TEAB) at 37 °C for 30 min and alkylated in an alkylating buffer (100 mM iodoacetamide in 100 mM TEAB) in the dark at room temperature for 30 min. After that, tryptic digestion was performed using Trypsin and gold (mass spectrometry grade; Promega, USA) for 16 h at 37 °C. The tryptic peptides were dried in the Nitrogen Evaporator (Organomation, USA) and stored at − 80 °C until further processing.

For Nano-LC–MS/MS analysis, the protein sample was resuspended in 0.1% formic acid, cleaned up by a C18 zip-tip, and analyzed using an LC–MS/MS system, including a Nano-liquid chromatograph (Dionex Ultimate 3000, RSLCnano System, Thermo Scientific) in combination with a CaptiveSpray source/Quadrupole ion trap mass spectrometer (Model Q-ToF Compact, Bruker, Germany). In brief, a microgram of peptides was enriched using a Nano trap column (C18 PepMap100) and Acclaim PepMap100 and then separated using the C18 PepMap100 LC column. Elution was performed using a linear gradient of 2–95% Solvent B (0.08% FA in 80% acetonitrile), and the column temperature was set at 60 °C. The loading pump solvent consisted of 0.05%TFA in 2% acetonitrile. The product was dried with a gas flow rate of 5 l/min. LC–MS was performed in a positive ionization mode with a survey scan mass range of m/z 150–2200, and mass spectral rates were performed at low (4 Hz) and high (16 Hz).

### Database processing and bioinformatics analysis

MaxQuant v1.6.2.10 software was used to quantify the proteins, and the Andromeda search engine was used to correlate MS/MS spectra with the UniProt *Homo sapiens* database. Label-free quantitation was performed using the standard MaxQuant settings: peptide tolerance for first and main searches set as 0.5 and 0.25, trypsin as the digestive enzyme, a maximum of two missed cleavages, oxidation of methionine and acetylation of the protein N-terminus as variable modifications, while carbamidomethylation of cysteine residues was set as a fixed modification. Raw data were blasted against the *Homo sapiens* database. The protein false discovery rate (FDR) was set at 1%. TOF MS/MS match tolerance was set at 0.5 Da with label-free quantification. A match between run options in the software was used for mass and retention time recalibrating between runs.

We utilized the PANTHER database, accessed in August 2024, to categorize the Gene Ontology in terms of molecular functions, biological processes, and pathways of the identified proteins in the AFSC-se. The STRING v12.0 database (https://string-db.org/) was used to annotate protein interactions, and the KEGG pathway was combined to indicate the functional pathway of proteins. We used Cytoscape’s ClueGO plug-in v3.10.2 to identify the top cluster of protein network interaction to specify therapeutic effects.

### Statistical evaluation

Data (the mean ± standard deviation; SD) were analyzed using Student’s *t*-test in PRISM software version 8.0 (GraphPad Software). Statistical significance was set at *p*-value < 0.05.

## Results

### Characteristics of AFSCs and secretory molecules

**Fig. 1:**
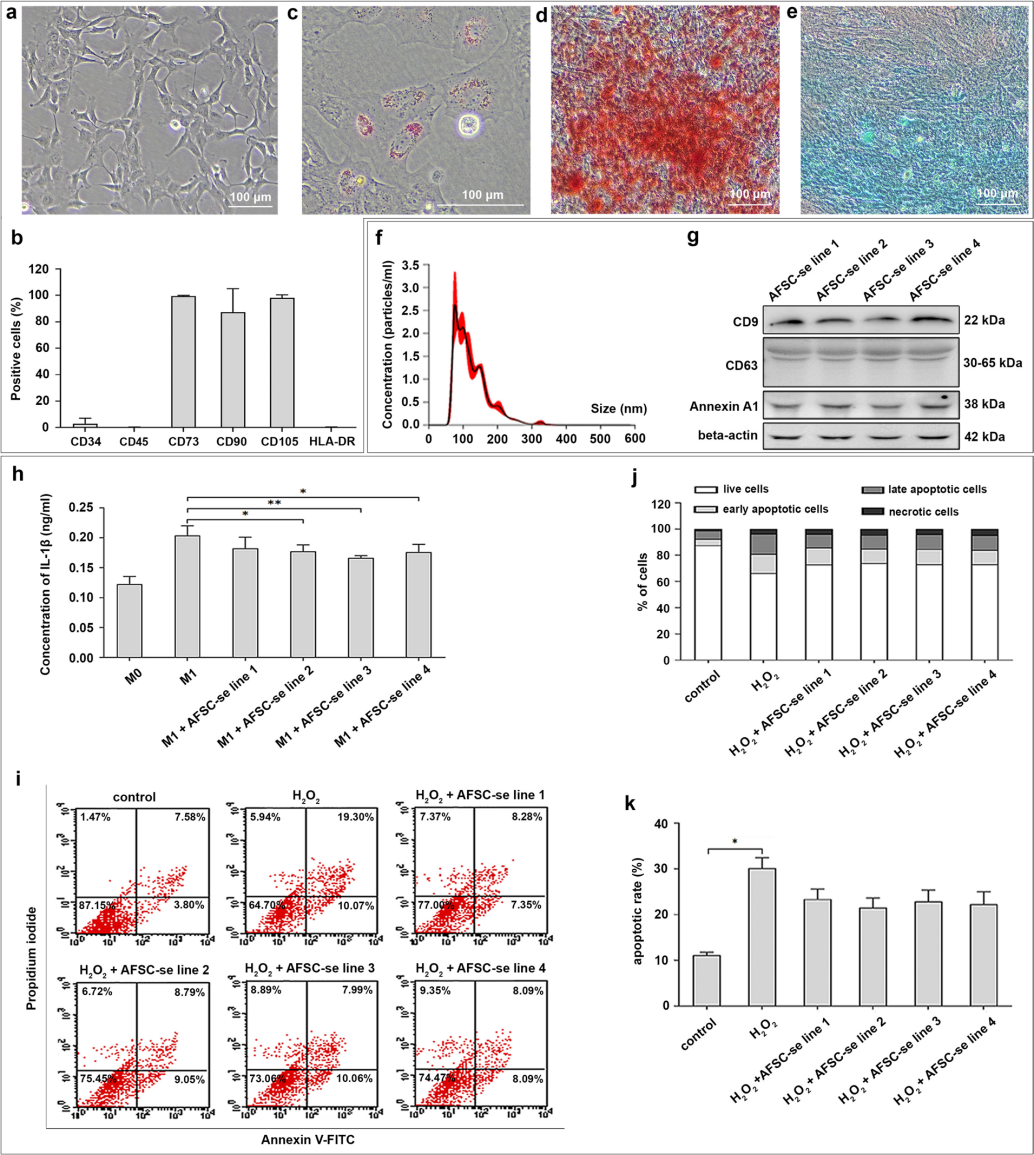
Characteristics of AFSC and secretory molecules. **a** The AFSC utilized in this study is presented in the cell morphology and **b** characteristics by specific protein markers on the cell surface. The appearance of lineage-specific signals for adipocytes is shown by **c** Oil Red O-stained lipid droplets, while osteoblasts are identified with **d** Alizarin red S staining and chondrocyte signals are indicated by **e** Alcian blue staining. The AFSC-se from four AFSC lines was screened. **f** Graphs generated by the NTA software depict particle concentration on the *Y*-axis and size on the *X*-axis. **g** Western blot analysis confirms the presence of EV markers, with full-length blots available in Supplementary Figure S1. The therapeutic effects of AFSC-se (*n* = 4) are demonstrated by anti-inflammatory properties, shown by reduced IL-1β production in **h** M1 macrophages, and antiapoptotic effects indicated by more viable cells after **i, j** H_2_O_2_ exposure. The apoptotic rate in H_2_O_2_-induced dermal fibroblasts is presented in panel **k**. All experiments were performed in triplicate; *p-value < 0.05 and **p-value < 0.01

Four AFSC lines in passage 7 used in this study consistently exhibited a spindle-shaped morphology (Fig. 1a). The cells had a proliferation rate ranging from 1.0 to 1.8 days, with an average population doubling time of 1.48 ± 0.3 days. They expressed MSC markers: CD34 −, CD45 −, CD73 +, CD90 +, CD105 +, and MHC class II (HLA-DR-) (Fig. 1b). All cell lines also demonstrated the ability to differentiate into adipocytes, osteocytes, and chondrocytes (Fig. 1c–e).

The secreted proteins of AFSCs (AFSC-se) were obtained from the culture medium of individual AFSC lines using a nano-filter with a 10 kDa cutoff to capture bioactive molecules. NTA revealed that molecular particles in four secretome samples ranged in size from 70 to 200 nm, with the majority having a diameter of 120 nm. The concentrations of four AFSC-se samples ranged from 1.1 × 10^10^ to 2.4 × 10^10^ particles/ml (Fig. 1f). The secretome samples were found to have the presence of CD9, CD63, and Annexin A1 on the identified particles (Fig. 1g).

### Therapeutic effects of AFSC-se

The AFSC-se from individual AFSC cell lines was assessed for therapeutic properties, including its ability to reduce inflammation and prevent cell death. The results showed that the secretome had a positive impact on reducing inflammation. In an in vitro model of THP-1 macrophage polarization, M1 treated with AFSC-se showed a substantial decrease in IL-1β production compared to untreated M1 (*p*-value = 0.0012) (Fig. 1h).

Our analysis also indicates that AFSC-se has the potential to prevent cell death. The percentage of live cells in the H_2_O_2_-supplemented medium decreased to 63.64% ± 1.83% compared to 87.56% ± 0.79% in the regular culture medium. However, the percentage of live cells was found to increase to an average of 73.36% ± 5.05% in H_2_O_2_-exposed cells co-treated with AFSC-se. Flow cytometry and calculation of apoptotic rate showed a higher presence of apoptotic cells in cells not treated with AFSC-se (Fig. 1i–k).

These analyses indicate that the AFSC-se possesses favorable therapeutic properties.

### AFSC-se proteome

The AFSC-se of four different lines of AFSC underwent proteomic analysis, resulting in four distinct datasets of proteomic profiles. This approach aimed to minimize variations in protein compositions among the different cell lines. The data obtained were processed using MaxQuant software and analyzed against the human UniProt database.

In the LC/MS–MS analysis, 1269 proteins were identified in AFSC-se line 1, 1230 proteins in AFSC-se line 2, 1059 proteins in AFSC-se line 3, and 1166 in AFSC-se line 4. After filtering out the co-expressed protein data among the four cell lines, we identified 2193 distinct proteins expressed across the four AFSC-se samples. These 2193 proteins were further analyzed using the PANTHER database to determine their biological processes, molecular functions, and pathways.

**Fig. 2:**
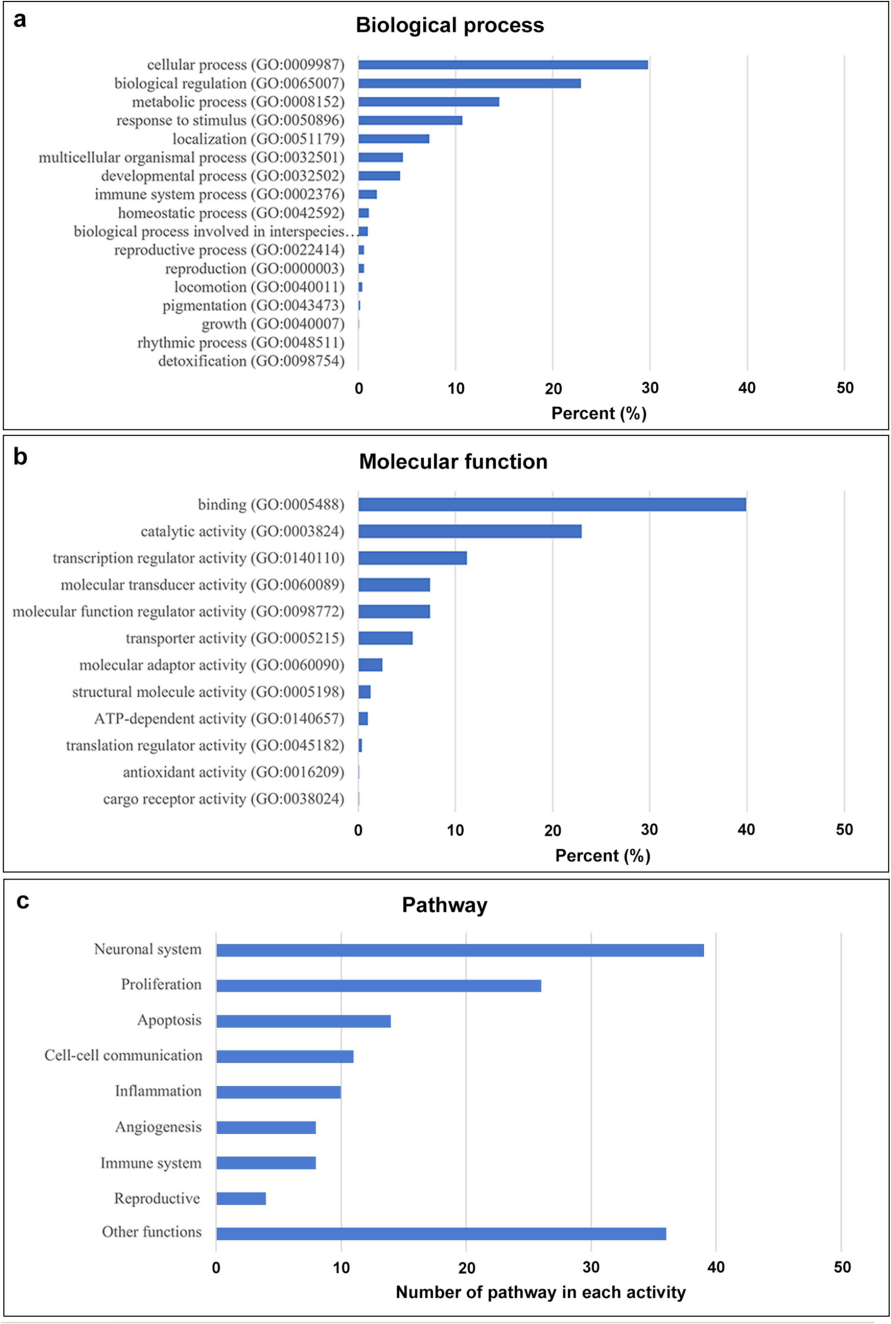
PANTHER analysis performed on a GO annotation of the identified proteins in AFSC-se: **a** biological process, **b** molecular function, and **c** pathway classification

In the context of biological processes, the PANTHER database classified 1402 out of 2193 proteins associated with 2765 biological processes. The top five biological processes are cellular process (825 proteins, 29.8%), biological regulation (631 proteins, 22.9%), metabolic process (400 proteins, 14.5%), response to stimulus (297 proteins, 10.7%), and localization (201 proteins, 7.3%) (Fig. 2a). In terms of molecular functions, 1306 out of 2193 proteins were identified, linked to 1577 functions. The top five functions include binding (629 proteins, 39.9%), catalytic activity (363 proteins, 23%), transcription regulator activity (176 proteins, 11.2%), molecular function regulator activity (117 proteins,7.4%), and molecular transducer activity (117 proteins, 7.4%) (Fig. 2b). As for the pathway of action, the AFSC secretory proteins are involved in at least 112 pathways of action. A complete list of these pathways can be found in the supplementary data (Table S1). The top five pathways were inflammation mediated by chemokine and cytokine signaling pathway (41 proteins, 4.5%), Wnt signaling pathway (38 proteins, 4.1%), gonadotropin-releasing hormone receptor pathway (30 proteins, 3.3%), CCKR signaling map (28 proteins, 3%), and heterotrimeric G-protein signaling pathway (23 proteins, 2.5%).

To better understand the functional role of AFSC-se, we can analyze and categorize the 112 pathways into nine distinct groups based on their activity and the significant number of pathways involved. Three of these groups are associated with specific organ systems, including the nervous system (39 pathways), the immune system (8 pathways), and the reproductive system (4 pathways). Additionally, four groups are associated with the therapeutic function of stem cells, which include proliferation (26 pathways), apoptosis (14 pathways), inflammation (10 pathways), and angiogenesis (8 pathways), as illustrated in Fig. 2c. This analysis provides an overview of the proteomic landscape and the functional role of bioactive molecules in AFSC-se.

### Top 250 proteins reveal prioritized functions impacted by AFSC-se

**Fig. 3:**
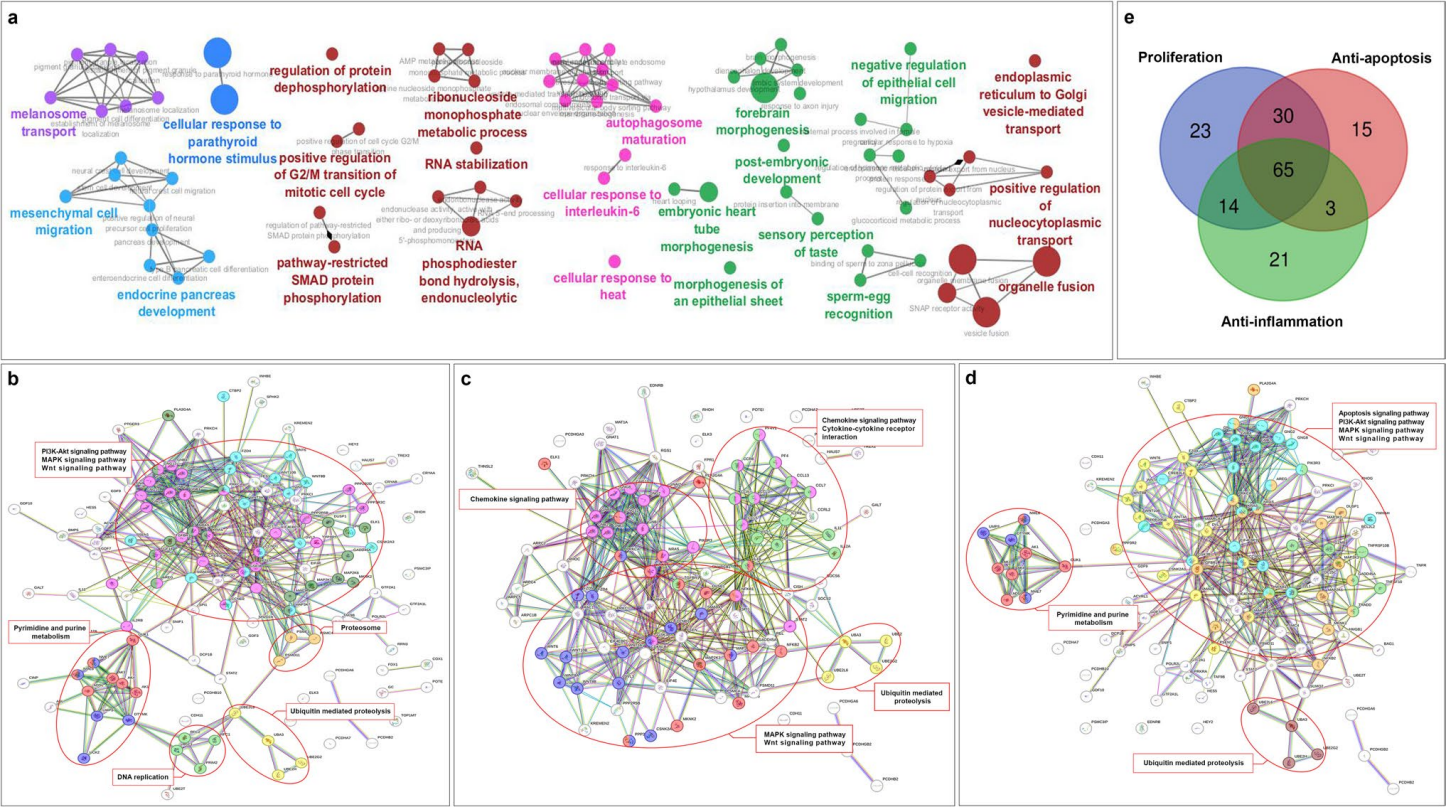
Protein networks. **a** ClueGO analysis classified the top 250 proteins of AFSC-se. The STRING database mapped the protein–protein interaction network for 132 proteins involved in **b** cell growth/proliferation, 103 proteins associated with **c** anti-inflammation activity, and 113 proteins linked to the **d** antiapoptosis mechanism in AFSC-se. Each node (colored circles) in the network represents all the proteins produced by a single protein-coding gene, including splicing isoforms and alternative polyadenylation forms. **e** Venn diagrams present the overlapping of 171 proteins in the function of proliferation, anti-inflammation, and antiapoptosis properties

To better understand the therapeutic effects of AFSC secretory molecules, we identified the top 250 proteins with the highest intensities. We analyzed their functions using the CluGo, focusing on the GO biological process (term *p*-value corrected with Bonferroni step-down, *p* ≤ 0.05). Our analysis revealed that the top three functions are organelle fusion, forebrain morphogenesis, and response to parathyroid hormone. Additionally, we categorized the top 250 proteins into four functional network groups: inflammation, transportation, cell growth, and development (Fig. 3a). A complete list of the top 250 proteins can be found in the supplementary data (Table S2).

We found that within a group of protein networks that function in tissue development, most protein networks are involved in the development of specific organs: (1) brain/neuron, (2) pancreas, and (3) heart. In the brain, there are 10 protein networks significantly present (*p*-value ≤ 0.05) and involved in brain development. Among these, the networks associated with forebrain morphogenesis (GO:0048853) and hypothalamus development (GO:0021854) are the most highly represented (*p*-value = 0.001). In the pancreas, there are 5 protein networks involved in endocrine pancreas development (*p*-value = 0.05). The most prevalent are proteins related to endocrine pancreas development (GO:0031018) and type B pancreatic cell differentiation (GO:0003309). Additionally, two protein networks are involved in heart development, with the highest presence in embryonic heart tube morphogenesis (GO:0003143).

The analysis of the top 250 proteins suggests that bioactive molecules of AFSC-se primarily affect growth and development, cell communication, and apoptosis.

### Proteins associate with therapeutic properties of MSCs

The fundamental therapeutic actions of MSCs involve cell growth/proliferation, anti-inflammation, and antiapoptosis. Our in vitro study discovered that AFSC-se can reduce the secretion of IL-1β from macrophages in an anti-inflammatory response (Fig. 1h) and increase the number of viable cells in an antiapoptotic manner (Fig. 1i–k). We then analyzed the proteins in AFSC-se associated with basic therapeutic functions using the STRING database and KEGG pathway.

Regarding cell growth/proliferation, the protein–protein interaction network consisted of 132 proteins, which included 130 nodes and 464 edges linking them. The significant interactions identified encompassed the PI3 K-Akt signaling pathway, MAPK signaling pathway, Wnt signaling pathway, proteasome, as well as pyrimidine and purine metabolism, DNA replication, and ubiquitin-mediated proteolysis (Fig. 3b). For anti-inflammation, the protein network comprised 103 proteins, with 101 nodes and 494 edges connecting them. Key interactions included the MAPK signaling pathway, Wnt signaling pathway, chemokine signaling pathway, cytokine-cytokine receptor interactions, and ubiquitin-mediated proteolysis (Fig. 3c). In terms of antiapoptosis, the protein network included 113 proteins, composed of 111 nodes and 440 edges linking them. Major interactions identified featured the apoptosis signaling pathway, PI3 K-Akt signaling pathway, MAPK signaling pathway, Wnt signaling pathway, pyrimidine and purine metabolism, and ubiquitin-mediated proteolysis (Fig. 3d).

In our observation, we discovered that 171 out of 2193 proteins (7.8%) in AFSC-se respond to the fundamental therapeutic properties of MSCs, specifically in cell growth/proliferation, reducing inflammation, and preventing apoptosis. Many of the proteins associated with these three characteristics overlap (Fig. 3e). Only 23 proteins demonstrated a unique role in cell growth/proliferation, 21 proteins demonstrated in only anti-inflammation, and 15 proteins in antiapoptosis, while 65 proteins were identified as involved in the effects of cell growth, anti-inflammation, and antiapoptosis. This overlap suggests that these three functions work synergistically and are fundamental to the therapeutic potential of AFSCs. These findings confirm the relationship between the proteomic profile and stem cell function, indicating that the proteomic profile could serve as a valuable indicator of stem cell activity for cell therapy.

### AFSC-se contains numerous proteins supporting brain function

**Fig. 4:**
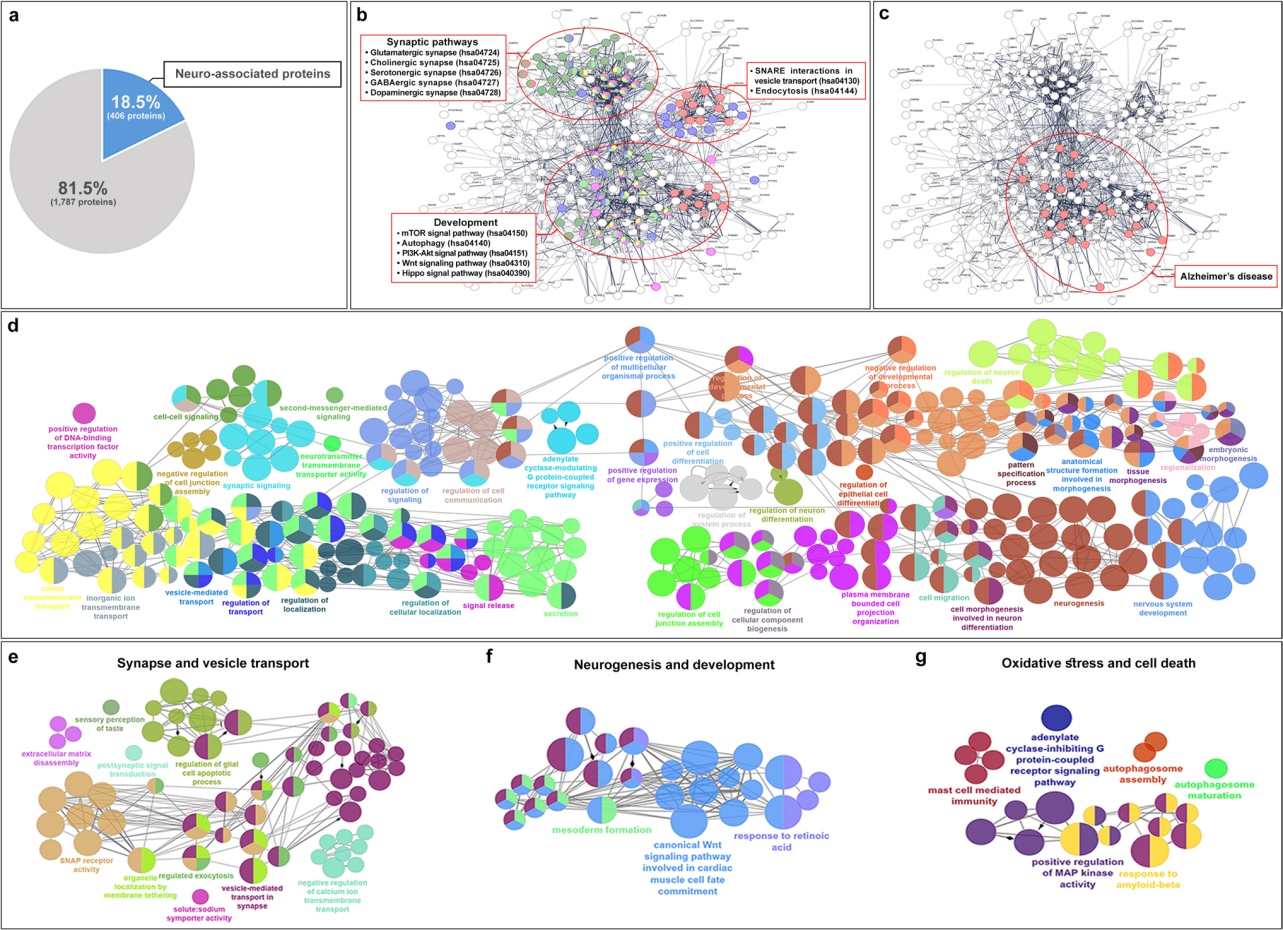
Proteomic network of the 406 neuro-associated proteins found in AFSC-se. **a** AFSC-se enriches protein-related neurodevelopment. STRING and KEGG pathways were mapped to classify the 406 neuro-associated proteins in AFSC-se based on the **b** function network and the **c** AD-associated protein network. ClueGO analysis demonstrates the therapeutic function of 406 neuro-associated proteins in AFSC-se, **d** including neuron regeneration, restoring function, and synaptogenesis. ClueGO analysis displays the therapeutic action of these 80 neuro-associated proteins in the context of Alzheimer’s disease in **e** synaptic and vesicle transportation, **f** neurogenesis and development, and **g** oxidative stress and cell death

We observed that AFSC-se contains a significant number of proteins associated with the brain. To investigate the functional role of proteins of AFSC-se in the brain, we analyzed 2193 identified proteins using the NCBI protein database, the Gene Ontology biological process, and the PANTHER database, focusing on proteins involved in neurological functions. Our results found 406 proteins (Table S3), 18.5% of the total associated with 39 pathways of action related to brain activity, neurological processes, and neural development (Fig. 4a).

These 406 proteins were mapped to create a protein interaction network using the STRING database, and their functions were analyzed by integrating KEGG pathway data. The analysis revealed three distinct protein interaction networks. The first network mainly consists of proteins involved in five synaptic pathways, while the second group includes proteins associated with SNARE interactions in vesicle transport. The third group predominantly features proteins linked to pathways related to cell genesis, cell survival, cell growth, and development (Fig. 4b). Notably, we found that proteins in the third network are linked to the context of various neurodegenerative diseases, such as Alzheimer’s (Fig. 4c).

To further understand the therapeutic functions of these 406 proteins, we conducted an analysis using ClueGO. The result indicated that these proteins primarily influence brain development and neuron activity. Their roles in brain development include neurogenesis, neural growth, cell migration, neuron death, and morphogenesis. The neuronal activity related to synaptic transmission involves cell–cell signaling, synaptic signaling, trafficking of synaptic vesicles, vesicle-mediated transport, transmembrane transport, and regulation of cell communication (Fig. 4d).

We identified 406 neuro-associated proteins linked to the context of Alzheimer’s disease (AD) through analyses using the UniProt and PANTHER databases. Among these, 80 proteins (19.7%) were specifically associated with the Alzheimer’s network. After analyzing the 80 AD-associated proteins with the STRING database and KEGG pathway data to assess their functional roles, we found that these proteins primarily engage in three activities: synaptic and vesicle transport, neurogenesis and development, and responses to oxidative stress and cell death (Fig. 4e–g).

Overall, our proteomic analysis of the 406 neuro-associated proteins indicates that AFSC-se contains numerous proteins with the potential to restore synaptic function and regenerate neural cells.

## Discussion

The therapeutic effects of MSCs are significantly influenced by the secretory molecules they release, which contribute to various significant therapeutic benefits (Gwam et al. 2021; Miceli et al. 2021). However, MSCs derived from different sources share only about 60% of similar proteins, leading to variations in their therapeutic capabilities. MSCs from amniotic fluid originate from fetal cells and exhibit an intermediate potency between embryonic and adult stem cells. This unique characteristic grants them superior proliferation abilities, making them the only type of MSCs to be utilized in autologous fetal therapy (Abe et al. 2023; Antounians et al. 2024). Our proteomic study has shown that AFSC-se contains a wide range of proteins that provide anti-inflammatory and antiapoptotic effects. This finding aligns with our in vitro study on the anti-inflammatory and antiapoptotic actions of AFSC-se. These results suggest that the proteins expressed in AFSC-se may serve as indicators of the therapeutic actions of amniotic fluid-derived MSCs.

We discovered that nearly 20% of the identified proteins in AFSC-se have a strong affinity for brain function and development. This percentage is higher than those findings in other types of MSCs (Gupta et al. 2022). This difference may be attributed to an epigenomic change of AFSCs, which are obtained from fetuses between 16 and 24 weeks of gestation, a period known for peak neurodevelopment. During this crucial time, the neocortex, responsible for higher brain functions, undergoes dynamic development (Stepien et al. 2021). The brain produces over 200,000 neurons every minute, with each neuron forming connections with thousands of other neurons. This process results in at least a trillion synapses in the human brain (Li and Tsien 2017). MSCs deriving from this stage are likely to provide significant support for neurodevelopment. It was also found that neuro-associated proteins secreted from AFSCs serve a similar functional role to those produced by primary neuron cells (Sharma et al. 2019). These factors operate within signaling networks to support neurogenesis, differentiation, and synaptogenesis. Furthermore, AFSCs display dynamic behavior and possess a secretory potential comparable to that of neural crest cells, originating from the neural tube during early brain development (Bronner and LeDouarin 2012; Dupin et al. 2007).

Our findings indicate that 1 out of every 5 proteins (406 out of 2193) secreted in the AFSC-se are associated with neurological functions. Among these 406 neuro-associated proteins, 40 are involved in the PI3 K-Akt and mTOR signaling pathways, promoting nerve cell proliferation and migration (Xie et al. 2021). The collaboration of these two pathways, often referred to as the PI3 K-Akt-mTOR signaling pathway, is crucial for managing neuro-protective action, including the regulation of neuroinflammation, neuronal oxidative stress, and neuroapoptosis (Iranpanah et al. 2023). The proteomic results also show that AFSC-se contains proteins that stimulate neuronal regeneration, promote synaptogenesis, and reduce oxidative stress. This supports previous reports on the effectiveness of AFSCs in treating nervous system disorders. They have demonstrated the ability to increase endogenous repair mechanisms in stroke models (Sibov et al. 2019), facilitate neurorestoration in cases of neonatal hypoxic-ischemic encephalopathy (Otani et al. 2019), and enhance improvements in spinal muscular atrophy (Shaw et al. 2021).

Additionally, AFSCs have been found to enhance higher brain functions, such as spatial cognition and short-term memory (Abe et al. 2023), and improve behavior in models of Parkinson's disease (Chang et al. 2013). They also protect neural function from oxidative stress and neuro-apoptosis in Alzheimer’s disease (AD) models as well (Zavatti et al. 2022). AD arises from disruptions in neural cell communication, primarily due to the toxic effects of beta-amyloid plaques and the interference caused by tau proteins, which form neurofibrillary tangles. This disruption ultimately leads to neural cell death. As a potential treatment for Alzheimer's disease, AFSC-derived proteins may offer significant benefits. Studies by Gatti et al. (2020) and Zavatti et al. (2022) have examined the effects of AFSC-EVs on mouse neurons affected by Alzheimer's. They discovered that AFSC-EVs exhibit antiapoptotic effects, leading to restored cell viability, reduced levels of annexin V, and activation of Akt, crucial molecules in the neuronal survival pathway. Furthermore, AFSC-EVs treatment decreased the expression of amyloid precursor proteins (APP) and phosphorylated tau (p-Tau), which may help halt the progression of Alzheimer’s disease. Moreover, AFSCs have shown success in treating various neurological disorders in multiple studies (Chang et al. 2013; Otani et al. 2019; Shaw et al. 2021; Sibov et al. 2019; Zavatti et al. 2022).

Our goal was to explore the bioactive molecules present in biological fluid that are released from AFSCs. We utilized the ultrafiltration technique to collect these secretory molecules by passing the fluid through a porous membrane. This method allowed us to concentrate the molecules within the biological fluid and isolate particles based on their molecular weight, adhering to the size specifications provided by the manufacturer. In this study, we employed a membrane specifically designed to capture secretory particles in the size range of 30 to 200 nm. This range encompasses exosomes, which are EVs known for their potential therapeutic effects. This technique enables us to obtain bioactive molecules containing EVs and soluble proteins, providing insight into the actual molecules secreted by AFSCs that are related to therapeutic effects.

## Conclusions

Proteomic analysis has revealed that AFSCs secrete a wide variety of proteins. Our work presents a relation between these proteins and the therapeutic effects of AFSC-se. There is a significant presence of neuro-associated proteins that are linked to neuroregeneration and neuroprotection. This suggests that AFSCs may be a promising candidate for the treatment of neurological diseases. Exploring the proteomics of MSCs not only helps to fill existing knowledge gaps in the field but also offers valuable insights into stem cell functionality, leading to improved MSC therapies in the future.

## Supplementary Information

Below is the link to the electronic supplementary material.

Supplementary file1 (DOCX 537 KB)

Supplementary file2 (DOCX 28 KB)

Supplementary file3 (DOCX 32 KB)

Supplementary file4 (DOCX 44 KB)

## Data Availability

The datasets generated during and/or analyzed during the current study are available in the Japan ProteOme Standard Repository (jPOSTrepo) with the dataset identifier PXD057833.

## References

[CR1] Abe Y, Sato Y, Tanaka M, Ochiai D (2023) Development of a new treatment for preterm birth complications using amniotic fluid stem cell therapy. Histol Histopathol 38:965–974 36971371 10.14670/HH-18-607

[CR2] Angulski AB, Capriglione LG, Batista M, Marcon BH, Senegaglia AC, Stimamiglio MA, Correa A (2017) The protein content of extracellular vesicles derived from expanded human umbilical cord blood-derived CD133^+^ and human bone marrow-derived mesenchymal stem cells partially explains why both sources are advantageous for regenerative medicine. Stem Cell Rev Rep 13:244–257 28054239 10.1007/s12015-016-9715-z

[CR3] Antounians L, Figueira RL, Kukreja B, Litvack ML, Zani-Ruttenstock E, Khalaj K, Montalva L, Doktor F, Obed M, Blundell M, Wu T, Chan C, Wagner R, Lacher M, Wilson MD, Post M, Kalish BT, Zani A (2024) Fetal hypoplastic lungs have multilineage inflammation that is reversed by amniotic fluid stem cell extracellular vesicle treatment. Sci Adv 10:eadn5405 39058789 10.1126/sciadv.adn5405 PMC11277482

[CR4] Balbi C, Lodder K, Costa A, Moimas S, Moccia F, van Herwaarden T, Rosti V, Campagnoli F, Palmeri A, De Biasio P, Santini F, Giacca M, Goumans MJ, Barile L, Smits AM, Bollini S (2019) Reactivating endogenous mechanisms of cardiac regeneration via paracrine boosting using the human amniotic fluid stem cell secretome. Int J Cardiol 287:87–95 30987834 10.1016/j.ijcard.2019.04.011

[CR5] Bronner ME, LeDouarin NM (2012) Development and evolution of the neural crest: an overview. Dev Biol 366:2–9 22230617 10.1016/j.ydbio.2011.12.042 PMC3351559

[CR6] Chang YJ, Ho TY, Wu ML, Hwang SM, Chiou TW, Tsai MS (2013) Amniotic fluid stem cells with low γ-interferon response showed behavioral improvement in Parkinsonism rat model. PLoS ONE 8:e76118 24098771 10.1371/journal.pone.0076118 PMC3786896

[CR7] Corcelli M, Hawkins K, Vlahova F, Hunjan A, Dowding K, De Coppi P, David AL, Peebles D, Gressens P, Hagberg H, Hristova M, Guillot PV (2018) Neuroprotection of the hypoxic-ischemic mouse brain by human CD117^+^CD90^+^CD105^+^ amniotic fluid stem cells. Sci Rep 8:2425 29402914 10.1038/s41598-018-20710-9 PMC5799160

[CR8] D’Arrigo D, Roffi A, Cucchiarini M, Moretti M, Candrian C, Filardo G (2019) Secretome and extracellular vesicles as new biological therapies for knee osteoarthritis: a systematic review. J Clin Med 8:1867 31689923 10.3390/jcm8111867 PMC6912212

[CR9] Dupin E, Calloni G, Real C, Gonçalves-Trentin A, Le Douarin NM (2007) Neural crest progenitors and stem cells. C R Biol 330:521–529 17631447 10.1016/j.crvi.2007.04.004

[CR10] Figueira RL, Khoshgoo N, Doktor F, Khalaj K, Islam T, Moheimani N, Blundell M, Antounians L, Post M, Zani A (2024) Antenatal administration of extracellular vesicles derived from amniotic fluid stem cells improves lung function in neonatal rats with congenital diaphragmatic hernia. J Pediatr Surg 59:1771–1777 38519389 10.1016/j.jpedsurg.2024.02.029

[CR11] Gatti M, Zavatti M, Beretti F, Giuliani D, Vandini E, Ottani A, Bertucci E, Maraldi T (2020) Oxidative stress in alzheimer’s disease: In vitro therapeutic effect of amniotic fluid stem cells extracellular vesicles. Oxid Med Cell Longev 2020:2785343 33193997 10.1155/2020/2785343 PMC7641262

[CR12] Goo J, Lee Y, Lee J, Kim IS, Jeong C (2024) Extracellular vesicles in therapeutics: a comprehensive review on applications, challenges, and clinical progress. Pharmaceutics 16:311 38543204 10.3390/pharmaceutics16030311 PMC10974516

[CR13] Gupta S, Krishnakumar V, Soni N, Rao EP, Banerjee A, Mohanty S (2022) Comparative proteomic profiling of small extracellular vesicles derived from iPSCs and tissue specific mesenchymal stem cells. Exp Cell Res 420:113354 36126717 10.1016/j.yexcr.2022.113354

[CR14] Gwam C, Mohammed N, Ma X (2021) Stem cell secretome, regeneration, and clinical translation: a narrative review. Ann Transl Med 9:70 33553363 10.21037/atm-20-5030 PMC7859812

[CR15] Han Y, Yang J, Fang J, Zhou Y, Candi E, Wang J, Hua D, Shao C, Shi Y (2022) The secretion profile of mesenchymal stem cells and potential applications in treating human diseases. Signal Transduct Target Ther 7:92 35314676 10.1038/s41392-022-00932-0 PMC8935608

[CR16] Iranpanah A, Kooshki L, Moradi SZ, Saso L, Fakhri S, Khan H (2023) The exosome-mediated PI3K/Akt/mTOR signaling pathway in neurological diseases. Pharmaceutics 15:1006 36986865 10.3390/pharmaceutics15031006 PMC10057486

[CR17] Katifelis H, Filidou E, Psaraki A, Yakoub F, Roubelakis MG, Tarapatzi G, Vradelis S, Bamias G, Kolios G, Gazouli M (2022) Amniotic fluid-derived mesenchymal stem/stromal cell-derived secretome and exosomes improve inflammation in human intestinal subepithelial myofibroblasts. Biomedicines 10:2357 36289619 10.3390/biomedicines10102357 PMC9598363

[CR18] Kou M, Huang L, Yang J, Chiang Z, Chen S, Liu J, Guo L, Zhang X, Zhou X, Xu X, Yan X, Wang Y, Zhang J, Xu A, Tse HF, Lian Q (2022) Mesenchymal stem cell-derived extracellular vesicles for immunomodulation and regeneration: a next generation therapeutic tool? Cell Death Dis 13:580 35787632 10.1038/s41419-022-05034-x PMC9252569

[CR19] Li M, Tsien JZ (2017) Neural code-neural self-information theory on how cell-assembly code rises from spike time and neuronal variability. Front Cell Neurosci 11:236 28912685 10.3389/fncel.2017.00236 PMC5582596

[CR20] Manni G, Gargaro M, Ricciuti D, Fontana S, Padiglioni E, Cipolloni M, Mazza T, Rosati J, di Veroli A, Mencarelli G, Pieroni B, Silva Barcelos EC, Scalisi G, Sarnari F, di Michele A, Pascucci L, de Franco F, Zelante T, Antognelli C, Cruciani G, Talesa VN, Romani R, Fallarino F (2024) Amniotic fluid stem cell-derived extracellular vesicles educate type 2 conventional dendritic cells to rescue autoimmune disorders in a multiple sclerosis mouse model. J Extracell Vesicles 13:e12446 38844736 10.1002/jev2.12446 PMC11156524

[CR21] Marassi V, La Rocca G, Placci A, Muntiu A, Vincenzoni F, Vitali A, Desiderio C, Maraldi T, Beretti F, Russo E, Miceli V, Conaldi PG, Papait A, Romele P, Cargnoni A, Silini AR, Alviano F, Parolini O, Giordani S, Zattoni A, Reschiglian P, Roda B (2024) Native characterization and QC profiling of human amniotic mesenchymal stromal cell vesicular fractions for secretome-based therapy. Talanta 276:126216 38761653 10.1016/j.talanta.2024.126216

[CR22] Miceli V, Bulati M, Iannolo G, Zito G, Gallo A, Conaldi PG (2021) Therapeutic properties of mesenchymal stromal/stem cells: the need of cell priming for cell-free therapies in regenerative medicine. Int J Mol Sci 22:763 33466583 10.3390/ijms22020763 PMC7828743

[CR23] Ning N, Wang Q, Li J, Liu B, Chen G, Hui J, An L (2023) Amniotic fluid stem cell attenuated necrotizing enterocolitis progression by promoting Rspo3/AMPKα axis. Immunobiology 228:152336 37173190 10.1016/j.imbio.2023.152336

[CR24] Otani T, Ochiai D, Masuda H, Abe Y, Fukutake M, Matsumoto T, Miyakoshi K, Tanaka M (2019) The neurorestorative effect of human amniotic fluid stem cells on the chronic phase of neonatal hypoxic-ischemic encephalopathy in mice. Pediatr Res 85:97–104 30120407 10.1038/s41390-018-0131-8

[CR25] Sato Y, Ochiai D, Abe Y, Masuda H, Fukutake M, Ikenoue S, Kasuga Y, Shimoda M, Kanai Y, Tanaka M (2020) Prophylactic therapy with human amniotic fluid stem cells improved survival in a rat model of lipopolysaccharide-induced neonatal sepsis through immunomodulation via aggregates with peritoneal macrophages. Stem Cell Res Ther 11:300 32690106 10.1186/s13287-020-01809-1 PMC7370504

[CR26] Sharma P, Mesci P, Carromeu C, McClatchy DR, Schiapparelli L, Yates JR 3rd, Muotri AR, Cline HT (2019) Exosomes regulate neurogenesis and circuit assembly. Proc Natl Acad Sci U S A 116:16086–16094 31320591 10.1073/pnas.1902513116 PMC6689941

[CR27] Shaw SW, Peng SY, Liang CC, Lin TY, Cheng PJ, Hsieh TT, Chuang HY, De Coppi P, David AL (2021) Prenatal transplantation of human amniotic fluid stem cell could improve clinical outcome of type III spinal muscular atrophy in mice. Sci Rep 11:9158 33911155 10.1038/s41598-021-88559-z PMC8080644

[CR28] Sibov TT, Pavon LF, Cabral FR, Cunha IF, de Oliveira DM, de Souza JG, Marti LC, da Cruz EF, Malheiros JM, Paiva FF, Tannús A, de Oliveira SM, da Costa MDS, Dastoli PA, Mendonça JN, de Toledo SRC, Malheiros SMF, de PaivaNeto MA, Rego NBB, Moron AF, Cavalheiro S (2019) Intravenous grafts of human amniotic fluid-derived stem cells reduce behavioral deficits in experimental ischemic stroke. Cell Transplant 28:1306–1320 31161782 10.1177/0963689719854342 PMC6767884

[CR29] Stepien BK, Vaid S, Huttner WB (2021) Length of the neurogenic period-a key determinant for the generation of upper-layer neurons during neocortex development and evolution. Front Cell Dev Biol 9:676911 34055808 10.3389/fcell.2021.676911 PMC8155536

[CR30] Xie Y, Chen Y, Zhu Y, Chen X, Lin T, Zhou D (2021) Adipose mesenchymal stem cell-derived exosomes enhance PC12 cell function through the activation of the PI3K/AKT pathway. Stem Cells Int 2021:2229477 34691190 10.1155/2021/2229477 PMC8536463

[CR31] Zavatti M, Beretti F, Casciaro F, Bertucci E, Maraldi T (2020) Comparison of the therapeutic effect of amniotic fluid stem cells and their exosomes on monoiodoacetate-induced animal model of osteoarthritis. BioFactors 46:106–117 31625201 10.1002/biof.1576

[CR32] Zavatti M, Gatti M, Beretti F, Palumbo C, Maraldi T (2022) Exosomes derived from human amniotic fluid mesenchymal stem cells preserve microglia and neuron cells from Aβ. Int J Mol Sci 23:4967 35563358 10.3390/ijms23094967 PMC9105787

[CR33] Zou Y, Liao L, Dai J, Mazhar M, Yang G, Wang H, Dechsupa N, Wang L (2023) Mesenchymal stem cell-derived extracellular vesicles/exosome: a promising therapeutic strategy for intracerebral hemorrhage. Regen Ther 22:181–190 36860266 10.1016/j.reth.2023.01.006 PMC9969203

