# Supplementary material for: Proteomic analysis of secreted proteins derived from amniotic fluid stem cells
Source: Cell Tissue Res. 2025 Jun 7;401(3):275–86. doi: 10.1007/s00441-025-03984-0 (PMC12411586; doi:10.1007/s00441-025-03984-0)
Supplement: Supplementary file 1 — Supplementary file1 (DOCX 537 KB) [file 441_2025_3984_MOESM1_ESM.docx]

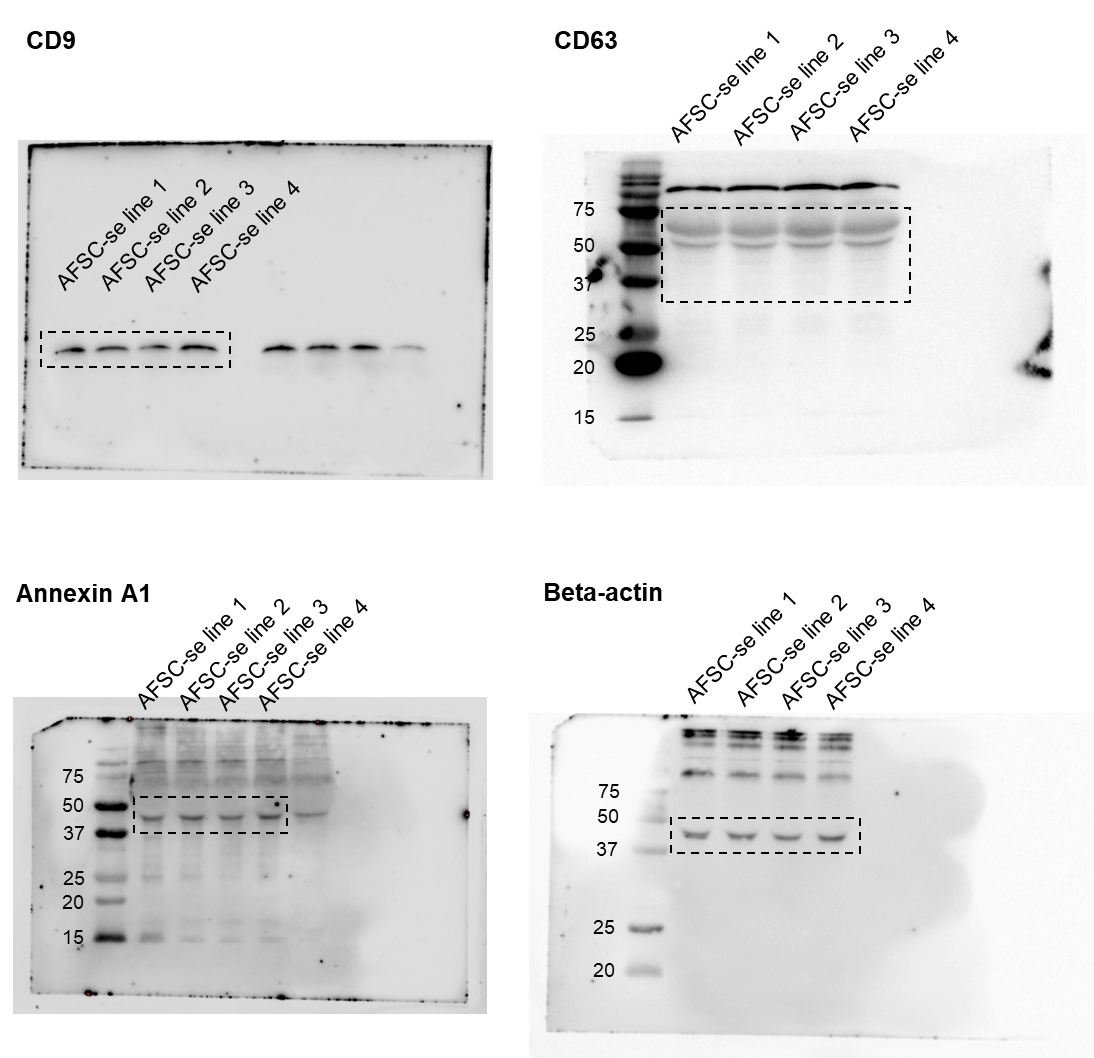


**Supplementary Figure S1.** Full length of Western blots in Fig. 1g. Dot boxes indicate the cropped blots shown in Fig. 1g.
