# Supplementary material for: Proteomic analysis of secreted proteins derived from amniotic fluid stem cells
Source: Cell Tissue Res. 2025 Jun 7;401(3):275–86. doi: 10.1007/s00441-025-03984-0 (PMC12411586; doi:10.1007/s00441-025-03984-0)
Supplement: Supplementary file 2 — Supplementary file2 (DOCX 28 KB) [file 441_2025_3984_MOESM2_ESM.docx]

**Table S1. Classification of the 112 pathways into 8 groups based on biological activity of AFSC-se**

| **Pathway names** | **Pathway accession** | **Protein numbers** |
| --- | --- | --- |
| **1. neuronal system (104 proteins)** | | |
| 5HT1 type receptor mediated signaling pathway | P04373 | 14 |
| 5HT2 type receptor mediated signaling pathway | P04374 | 15 |
| 5HT3 type receptor mediated signaling pathway | P04375 | 3 |
| 5HT4 type receptor mediated signaling pathway | P04376 | 11 |
| Adrenaline and noradrenaline biosynthesis | P00001 | 7 |
| Alzheimer disease-amyloid secretase pathway | P00003 | 4 |
| Alzheimer disease-presenilin pathway | P00004 | 15 |
| Asparagine and aspartate biosynthesis | P02730 | 1 |
| Axon guidance mediated by netrin | P00009 | 4 |
| Axon guidance mediated by semaphorins | P00007 | 3 |
| Axon guidance mediated by Slit/Robo | P00008 | 3 |
| Bupropion degradation | P05729 | 1 |
| Cortocotropin releasing factor receptor signaling pathway | P04380 | 11 |
| Dopamine receptor mediated signaling pathway | P05912 | 15 |
| Endogenous cannabinoid signaling | P05730 | 7 |
| Enkephalin release | P05913 | 10 |
| GABA-B receptor II signaling | P05731 | 7 |
| Histamine H1 receptor mediated signaling pathway | P04385 | 11 |
| Histamine H2 receptor mediated signaling pathway | P04386 | 8 |
| Huntington disease | P00029 | 10 |
| Insulin/IGF pathway-protein kinase B signaling cascade | P00033 | 4 |
| Ionotropic glutamate receptor pathway | P00037 | 3 |
| Metabotropic glutamate receptor group II pathway | P00040 | 15 |
| Metabotropic glutamate receptor group III pathway | P00039 | 17 |
| Muscarinic acetylcholine receptor 1 and 3 signaling  pathway | P00042 | 16 |
| Muscarinic acetylcholine receptor 2 and 4 signaling  pathway | P00043 | 18 |
| Nicotine pharmacodynamics pathway | P06587 | 5 |
| Nicotinic acetylcholine receptor signaling pathway | P00044 | 10 |
| Opioid prodynorphin pathway | P05916 | 13 |
| Opioid proenkephalin pathway | P05915 | 12 |
| Opioid proopiomelanocortin pathway | P05917 | 12 |
| Oxytocin receptor mediated signaling pathway | P04391 | 13 |
| Parkinson disease | P00049 | 14 |
| Proline biosynthesis | P02768 | 1 |
| Synaptic vesicle trafficking | P05734 | 4 |
| Thyrotropin-releasing hormone receptor signaling  pathway | P04394 | 14 |
| Vasopressin synthesis | P04395 | 2 |
| Vitamin B6 metabolism | P02787 | 1 |
| Wnt signaling pathway | P00057 | 38 |

| **Pathway names** | **Pathway accession** | **Protein numbers** |
| --- | --- | --- |
| **2. proliferation (132 proteins)** | | |
| Arginine biosynthesis | P02728 | 1 |
| Cadherin signaling pathway | P00012 | 20 |
| Cell cycle | P00013 | 5 |
| De novo purine biosynthesis | P02738 | 7 |
| De novo pyrimidine deoxyribonucleotide biosynthesis | P02739 | 2 |
| De novo pyrimidine ribonucleotides biosythesis | P02740 | 2 |
| DNA replication | P00017 | 5 |
| EGF receptor signaling pathway | P00018 | 14 |
| Endothelin signaling pathway | P00019 | 6 |
| FGF signaling pathway | P00021 | 15 |
| General transcription by RNA polymerase I | P00022 | 2 |
| General transcription regulation | P00023 | 4 |
| Insulin/IGF pathway-mitogen activated protein kinase  kinase/MAP kinase cascade | P00032 | 2 |
| Interleukin signaling pathway | P00036 | 16 |
| JAK/STAT signaling pathway | P00038 | 1 |
| Notch signaling pathway | P00045 | 3 |
| p38 MAPK pathway | P05918 | 10 |
| PI3 kinase pathway | P00048 | 16 |
| Ras Pathway | P04393 | 9 |
| Salvage pyrimidine ribonucleotides | P02775 | 1 |
| TGF-beta signaling pathway | P00052 | 14 |
| Transcription regulation by bZIP transcription factor | P00055 | 6 |
| Ubiquitin proteasome pathway | P00060 | 9 |
| VEGF signaling pathway | P00056 | 12 |
| Vitamin D metabolism and pathway | P04396 | 3 |
| Wnt signaling pathway | P00057 | 38 |
| **3. apoptosis (113 proteins)** | | |
| Apoptosis signaling pathway | P00006 | 16 |
| De novo purine biosynthesis | P02738 | 7 |
| De novo pyrimidine deoxyribonucleotide biosynthesis | P02739 | 2 |
| De novo pyrimidine ribonucleotides biosynthesis | P02740 | 2 |
| EGF receptor signaling pathway | P00018 | 14 |
| Endothelin signaling pathway | P00019 | 6 |
| JAK/STAT signaling pathway | P00038 | 1 |
| Notch signaling pathway | P00045 | 3 |
| p38 MAPK pathway | P05918 | 10 |
| p53 pathway | P00059 | 11 |
| TGF-beta signaling pathway | P00052 | 14 |
| Transcription regulation by bZIP transcription factor | P00055 | 6 |
| Ubiquitin proteasome pathway | P00060 | 9 |
| Wnt signaling pathway | P00057 | 38 |

| **Pathway names** | **Pathway accession** | **Protein numbers** |
| --- | --- | --- |
| **4. cell-cell communication (120 proteins)** | | |
| Apoptosis signaling pathway | P00006 | 16 |
| B cell activation | P00010 | 9 |
| CCKR signaling map | P06959 | 28 |
| Cadherin signaling pathway | P00012 | 20 |
| EGF receptor signaling pathway | P00018 | 14 |
| Inflammation mediated by chemokine and cytokine  signaling pathway | P00031 | 41 |
| Interleukin signaling pathway | P00036 | 16 |
| JAK/STAT signaling pathway | P00038 | 1 |
| PDGF signaling pathway | P00047 | 11 |
| Parkinson disease | P00049 | 14 |
| Toll receptor signaling pathway | P00054 | 7 |
| **5. inflammation (104 proteins)** | | |
| Endothelin signaling pathway | P00019 | 6 |
| Inflammation mediated by chemokine and cytokine  signaling pathway | P00031 | 41 |
| Interferon-gamma signaling pathway | P00035 | 3 |
| Interleukin signaling pathway | P00036 | 16 |
| JAK/STAT signaling pathway | P00038 | 1 |
| p38 MAPK pathway | P05918 | 10 |
| S-adenosylmethionine biosynthesis | P02773 | 1 |
| Ubiquitin proteasome pathway | P00060 | 9 |
| Vitamin B6 metabolism | P02787 | 1 |
| Wnt signaling pathway | P00057 | 38 |
| **6. angiogenesis (17 proteins)** | | |
| Angiogenesis | P00005 | 16 |
| VEGF signaling pathway | P00056 | 12 |
| **7. immune system (31 proteins)** | | |
| Arginine biosynthesis | P02728 | 1 |
| B cell activation | P00010 | 9 |
| Histamine H2 receptor mediated signaling pathway | P04386 | 8 |
| JAK/STAT signaling pathway | P00038 | 1 |
| S-adenosylmethionine biosynthesis | P02773 | 1 |
| T cell activation | P00053 | 7 |
| Toll receptor signaling pathway | P00054 | 7 |
| Vitamin D metabolism and pathway | P04396 | 3 |
| **8. reproduction (37 proteins)** | | |
| General transcription by RNA polymerase I | P00022 | 2 |
| General transcription regulation | P00023 | 4 |
| Gonadotropin-releasing hormone receptor pathway | P06664 | 30 |
| Transcription regulation by bZIP transcription factor | P00055 | 6 |

| **Pathway names** | **Pathway accession** | **Protein numbers** |
| --- | --- | --- |
| **9. other functions (100 proteins)** | | |
| Adenine and hypoxanthine salvage pathway | P02723 | 1 |
| Alpha adrenergic receptor signaling pathway | P00002 | 4 |
| Androgen/estrogene/progesterone biosynthesis | P02727 | 4 |
| Angiotensin II-stimulated signaling through G proteins and  beta-arrestin | P05911 | 11 |
| Beta1 adrenergic receptor signaling pathway | P04377 | 10 |
| Beta2 adrenergic receptor signaling pathway | P04378 | 10 |
| Beta3 adrenergic receptor signaling pathway | P04379 | 10 |
| Blood coagulation | P00011 | 3 |
| Carnitine metabolism | P02733 | 2 |
| Cholesterol biosynthesis | P00014 | 3 |
| Coenzyme A biosynthesis | P02736 | 2 |
| Coenzyme A linked carnitine metabolism | P02732 | 2 |
| Cysteine biosynthesis | P02737 | 1 |
| Cytoskeletal regulation by Rho GTPase | P00016 | 7 |
| Flavin biosynthesis | P02741 | 1 |
| Fructose galactose metabolism | P02744 | 2 |
| Glycolysis | P00024 | 3 |
| Hedgehog signaling pathway | P00025 | 2 |
| Heme biosynthesis | P02746 | 2 |
| Heterotrimeric G-protein signaling pathway-Gi alpha and  Gs alpha mediated pathway | P00026 | 23 |
| Heterotrimeric G-protein signaling pathway-Gq alpha and  Go alpha mediated pathway | P00027 | 21 |
| Heterotrimeric G-protein signaling pathway-rod outer  segment phototransduction | P00028 | 8 |
| Hypoxia response via HIF activation | P00030 | 3 |
| Integrin signaling pathway | P00034 | 14 |
| Mannose metabolism | P02752 | 1 |
| Methylmalonyl pathway | P02755 | 2 |
| N-acetylglucosamine metabolism | P02756 | 2 |
| Ornithine degradation | P02758 | 1 |
| Oxidative stress response | P00046 | 15 |
| p53 pathway by glucose deprivation | P04397 | 2 |
| p53 pathway feedback loops 2 | P04398 | 5 |
| Succinate to propionate conversion | P02777 | 1 |
| TCA cycle | P00051 | 1 |
| Thiamin metabolism | P02780 | 1 |
| Threonine biosynthesis | P02781 | 1 |
| Xanthine and guanine salvage pathway | P02788 | 1 |
