# Supplementary material for: Proteomic analysis of secreted proteins derived from amniotic fluid stem cells
Source: Cell Tissue Res. 2025 Jun 7;401(3):275–86. doi: 10.1007/s00441-025-03984-0 (PMC12411586; doi:10.1007/s00441-025-03984-0)
Supplement: Supplementary file 3 — Supplementary file3 (DOCX 32 KB) [file 441_2025_3984_MOESM3_ESM.docx]

**Table S2. List of top 250 proteins in AFSC-se**

| **Accession** | **Protein names** |
| --- | --- |
| P22003 | Bone morphogenetic protein 5 |
| Q49A33 | Putative zinc finger protein 876 |
| Q9H492 | Microtubule-associated proteins 1A/1B light chain 3A |
| Q9HB31 | Homeobox protein SEBOX |
| Q12887 | Protoheme IX farnesyltransferase, mitochondrial |
| P24539 | ATP synthase F(0) complex subunit B1, mitochondrial |
| O76061 | Stanniocalcin-2 |
| Q8IU54 | Interferon lambda-1 |
| Q9H3R5 | Centromere protein H |
| Q8NCB2 | CaM kinase-like vesicle-associated protein |
| Q9P086 | Mediator of RNA polymerase II transcription subunit 11 |
| Q7RTY3 | Serine protease 45 |
| P51810 | G-protein coupled receptor 143 |
| Q8IW40 | Coiled-coil domain-containing protein 103 |
| Q5JPI3 | Uncharacterized protein C3orf38 |
| Q5VV17 | OTU domain-containing protein 1 |
| O75131 | Copine-3 |
| Q9UQN3 | Charged multivesicular body protein 2b |
| O00212 | Rho-related GTP-binding protein RhoD |
| Q9BSF8 | BTB/POZ domain-containing protein 10 |
| Q9Y235 | Probable C->U-editing enzyme APOBEC-2 |
| P58876 | Histone H2B type 1-D |
| P31943 | Heterogeneous nuclear ribonucleoprotein H;Heterogeneous nuclear ribonucleoprotein H, N-terminally processed |
| Q9UNE7 | E3 ubiquitin-protein ligase CHIP |
| Q9P215 | Pogo transposable element with KRAB domain |
| O60383 | Growth/differentiation factor 9 |
| Q15722 | Leukotriene B4 receptor 1 |
| Q96PX6 | Coiled-coil domain-containing protein 85A |
| P18615 | Negative elongation factor E |
| Q96NU7 | Probable imidazolonepropionase |
| Q9BZD7 | Transmembrane gamma-carboxyglutamic acid protein 3 |
| Q96FV0 | Leucine-rich repeat-containing protein 46 |
| Q8TEY5 | Cyclic AMP-responsive element-binding protein 3-like protein 4;Processed cyclic AMP-responsive element-binding protein 3-like protein 4 |
| Q8N7R7 | Cyclin-Y-like protein 1 |
| Q58DX5 | Inactive N-acetylated-alpha-linked acidic dipeptidase-like protein 2 |
| P49368 | T-complex protein 1 subunit gamma |
| P08134 | Rho-related GTP-binding protein RhoC |
| Q6ZSU1 | Putative inactive cytochrome P450 2G1 |
| Q99895 | Chymotrypsin-C |
| Q86YC3 | Negative regulator of reactive oxygen species |
| Q92185 | Alpha-N-acetylneuraminide alpha-2,8-sialyltransferase |
| Q9H9S3 | Protein transport protein Sec61 subunit alpha isoform 2 |
| Q9HBG4 | V-type proton ATPase 116 kDa subunit a isoform 4 |
| Q96I34 | Protein phosphatase 1 regulatory subunit 16A |
| **Accession** | **Protein names** |
| Q9BQ50 | Three prime repair exonuclease 2 |
| Q9NPD8 | Ubiquitin-conjugating enzyme E2 T |
| Q96AJ9 | Vesicle transport through interaction with t-SNAREs homolog 1A |
| Q92543 | Sorting nexin-19 |
| Q96DX8 | Receptor-transporting protein 4 |
| Q330K2 | NADH dehydrogenase (ubiquinone) complex I, assembly factor 6 |
| Q8N0U7 | Uncharacterized protein C1orf87 |
| Q9NP64 | Nucleolar protein of 40 kDa |
| Q9NX45 | Spermatogenesis- and oogenesis-specific basic helix-loop-helix-containing protein 2 |
| Q13424 | Alpha-1-syntrophin |
| Q8TCB6 | Olfactory receptor 51E1 |
| P0DKX4 | Small integral membrane protein 18 |
| Q9UHK6 | Alpha-methylacyl-CoA racemase |
| Q5JVS0 | Intracellular hyaluronan-binding protein 4 |
| P17252 | Protein kinase C alpha type |
| Q96BZ9 | TBC1 domain family member 20 |
| Q92536 | Y+L amino acid transporter 2 |
| Q9NXP7 | Gypsy retrotransposon integrase-like protein 1 |
| Q8NHB7 | Olfactory receptor 5K1;Olfactory receptor 5K2 |
| P60852 | Zona pellucida sperm-binding protein 1;Processed zona pellucida sperm-binding protein 1 |
| P78346 | Ribonuclease P protein subunit p30 |
| Q8TC92 | Ecto-NOX disulfide-thiol exchanger 1;Hydroquinone [NADH] oxidase;Protein disulfide-thiol oxidoreductase |
| Q96IZ5 | RNA-binding protein 41 |
| P00492 | Hypoxanthine-guanine phosphoribosyltransferase |
| P52566 | Rho GDP-dissociation inhibitor 2 |
| Q9UL16 | Cilia- and flagella-associated protein 45 |
| P35520 | Cystathionine beta-synthase |
| Q8IUZ0 | Leucine-rich repeat-containing protein 49 |
| O75360 | Homeobox protein prophet of Pit-1 |
| Q9UNK0 | Syntaxin-8 |
| Q9Y3C1 | Nucleolar protein 16 |
| Q8TCG1 | Protein CIP2A |
| Q6NWY9 | Pre-mRNA-processing factor 40 homolog B |
| O60499 | Syntaxin-10 |
| Q9NVT9 | Armadillo repeat-containing protein 1 |
| Q8N187 | Calcium-responsive transcription factor |
| Q8N954 | G patch domain-containing protein 11 |
| Q9H633 | Ribonuclease P protein subunit p21 |
| Q8N309 | Leucine-rich repeat-containing protein 43 |
| Q5EBL2 | Zinc finger protein 628 |
| Q96PY0 | Putative uncharacterized protein PSMG3-AS1 |
| Q9NSE2 | Cytokine-inducible SH2-containing protein |

| **Accession** | **Protein names** |
| --- | --- |
| P80162 | C-X-C motif chemokine 6;Small-inducible cytokine B6, N-processed variant 1;Small-inducible cytokine B6, N-processed variant 2;Small-inducible cytokine B6, N-processed variant 3 |
| O75486 | Transcription initiation protein SPT3 homolog |
| Q8N108 | Mesoderm induction early response protein 1 |
| Q8IYX8 | Centrosomal protein CEP57L1 |
| P51864 | Putative teratocarcinoma-derived growth factor 3 |
| P08048 | Zinc finger Y-chromosomal protein |
| Q96S79 | Ras-like protein family member 10B |
| O60732 | Melanoma-associated antigen C1 |
| Q86Y29 | B melanoma antigen 3 |
| Q96PU5 | E3 ubiquitin-protein ligase NEDD4-like |
| Q8TAS1 | Serine/threonine-protein kinase Kist |
| Q96PQ1 | Sialic acid-binding Ig-like lectin 12 |
| Q8N5S1 | Solute carrier family 25 member 41 |
| Q52M93 | Zinc finger protein 585B |
| P04746 | Pancreatic alpha-amylase;Alpha-amylase 2B |
| Q9Y264 | Angiopoietin-4 |
| O15209 | Zinc finger and BTB domain-containing protein 22 |
| Q6V0L0 | Cytochrome P450 26C1 |
| Q9Y3E7 | Charged multivesicular body protein 3 |
| Q15717 | ELAV-like protein 1 |
| O43761 | Synaptogyrin-3 |
| O00311 | Cell division cycle 7-related protein kinase |
| Q9ULC3 | Ras-related protein Rab-23 |
| O95415 | Brain protein I3 |
| P57053 | Histone H2B type F-S;Histone H2B type 1-L;Histone H2B type 1-M;Histone H2B type 1-N;Histone H2B type 1-H;Histone H2B type 3-B;Histone H2B type 2-F;Histone H2B type 2-E;Histone H2B type 1-C/E/F/G/I;Histone H2B type 1-B;Histone H2B type 1-O;Histone H2B type 1-J;Histone H2B type 1-K |
| Q969X5 | Endoplasmic reticulum-Golgi intermediate compartment protein 1 |
| P51608 | Methyl-CpG-binding protein 2 |
| O15143 | Actin-related protein 2/3 complex subunit 1B |
| P52742 | Zinc finger protein 135 |
| Q96MC5 | Uncharacterized protein C16orf45 |
| P27144 | Adenylate kinase 4, mitochondrial |
| Q8NGN0 | Olfactory receptor 4D5 |
| P25106 | Atypical chemokine receptor 3 |
| Q8TCA0 | Leucine-rich repeat-containing protein 20 |
| Q96FV2 | Secernin-2 |
| Q9GZU5 | Nyctalopin |
| Q92688 | Acidic leucine-rich nuclear phosphoprotein 32 family member B |
| Q9Y278 | Heparan sulfate glucosamine 3-O-sulfotransferase 2 |
| Q8TCE6 | Protein FAM45A |
| Q9Y6C2 | EMILIN-1 |
| Q93045 | Stathmin-2 |
| P11926 | Ornithine decarboxylase |

| **Accession** | **Protein names** |
| --- | --- |
| P0C6T2 | Dolichyl-diphosphooligosaccharide--protein glycosyltransferase subunit 4 |
| Q32NC0 | UPF0711 protein C18orf21 |
| Q9ULS5 | Transmembrane and coiled-coil domains protein 3 |
| Q96D15 | Reticulocalbin-3 |
| Q00169 | Phosphatidylinositol transfer protein alpha isoform |
| Q9ULW3 | Activator of basal transcription 1 |
| Q8N8L2 | Zinc finger protein 491 |
| P56693 | Transcription factor SOX-10 |
| P23786 | Carnitine O-palmitoyltransferase 2, mitochondrial |
| Q8TDV0 | Probable G-protein coupled receptor 151 |
| Q9Y276 | Mitochondrial chaperone BCS1 |
| Q3ZCQ3 | Membrane protein FAM174B |
| Q9NRX1 | RNA-binding protein PNO1 |
| Q96IG2 | F-box/LRR-repeat protein 20 |
| Q6HA08 | Astacin-like metalloendopeptidase |
| Q9UN72 | Protocadherin alpha-7 |
| Q96QT4 | Transient receptor potential cation channel subfamily M member 7 |
| Q9HAA7 | Putative uncharacterized protein FLJ11871 |
| Q9BT73 | Proteasome assembly chaperone 3 |
| Q99835 | Smoothened homolog |
| Q6ZPA2 | Putative uncharacterized protein FLJ26174 |
| O95409 | Zinc finger protein ZIC 2 |
| P42331 | Rho GTPase-activating protein 25 |
| Q9ULX9 | Transcription factor MafF |
| Q969I6 | Sodium-coupled neutral amino acid transporter 4 |
| Q96CM8 | Acyl-CoA synthetase family member 2, mitochondrial |
| Q8IVL5 | Prolyl 3-hydroxylase 2 |
| Q9HBB8 | Cadherin-related family member 5 |
| Q86UF2 | cTAGE family member 6 |
| Q9NQZ5 | StAR-related lipid transfer protein 7, mitochondrial |
| P49711 | Transcriptional repressor CTCF |
| Q9UGB7 | Inositol oxygenase |
| Q9NPH6 | Odorant-binding protein 2b |
| Q8WUX2 | Putative glutathione-specific gamma-glutamylcyclotransferase 2 |
| Q9BXJ3 | Complement C1q tumor necrosis factor-related protein 4 |
| Q8TAI7 | GTPase RhebL1 |
| O43246 | Cationic amino acid transporter 4 |
| P60484 | Phosphatidylinositol 3,4,5-trisphosphate 3-phosphatase and dual-specificity protein phosphatase PTEN |
| Q9H4A4 | Aminopeptidase B |
| Q9NS98 | Semaphorin-3G |
| Q3SXZ3 | Zinc finger protein 718 |
| Q9GZV9 | Fibroblast growth factor 23;Fibroblast growth factor 23 N-terminal peptide;Fibroblast growth factor 23 C-terminal peptide |
| Q7L2R6 | Zinc finger protein 765 |
| P04899 | Guanine nucleotide-binding protein G(i) subunit alpha-2 |
| Q8N565 | Melanoregulin |
| **Accession** | **Protein names** |
| P29536 | Leiomodin-1 |
| Q9BQG2 | Peroxisomal NADH pyrophosphatase NUDT12 |
| Q8NH67 | Olfactory receptor 52I2 |
| Q9BXJ8 | Transmembrane protein 120A |
| P14784 | Interleukin-2 receptor subunit beta |
| Q6ZT89 | Solute carrier family 25 member 48 |
| P00746 | Complement factor D |
| O43169 | Cytochrome b5 type B |
| Q5SQH8 | Uncharacterized protein C6orf136 |
| P50591 | Tumor necrosis factor ligand superfamily member 10 |
| Q8IYJ1 | Copine-9 |
| Q8HWS3 | DNA-binding protein RFX6 |
| Q96L94 | Sorting nexin-22 |
| Q9GZK6 | Olfactory receptor 2J1 |
| Q92600 | Cell differentiation protein RCD1 homolog |
| Q9P291 | Armadillo repeat-containing X-linked protein 1 |
| Q96GI7 | Protein FAM89A |
| Q13938 | Calcyphosin |
| Q99467 | CD180 antigen |
| Q8N9B4 | Ankyrin repeat domain-containing protein 42 |
| Q3B820 | Protein FAM161A |
| Q5VWP2 | Protein FAM46C |
| Q9WJR5 | Endogenous retrovirus group K member 19 Pol protein;Reverse transcriptase;Ribonuclease H;Integrase |
| Q9NVN8 | Guanine nucleotide-binding protein-like 3-like protein |
| Q96JC9 | ELL-associated factor 1 |
| Q16585 | Beta-sarcoglycan |
| Q9H0A3 | Transmembrane protein 191A |
| Q9H9V9 | JmjC domain-containing protein 4 |
| P61244 | Protein max |
| Q8N0U4 | Protein FAM185A |
| P57737 | Coronin-7 |
| Q96CB5 | Putative uncharacterized protein C8orf44 |
| Q96NT3 | Protein GUCD1 |
| P53672 | Beta-crystallin A2 |
| Q8N5Y2 | Male-specific lethal 3 homolog |
| O43570 | Carbonic anhydrase 12 |
| Q5TGI0 | Failed axon connections homolog |
| Q8TDH9 | Biogenesis of lysosome-related organelles complex 1 subunit 5 |
| P39687 | Acidic leucine-rich nuclear phosphoprotein 32 family member A |
| Q96EL2 | 28S ribosomal protein S24, mitochondrial |
| O00744 | Protein Wnt-10b |
| Q9H840 | Gem-associated protein 7 |
| Q16553 | Lymphocyte antigen 6E |
| Q8WVK2 | U4/U6.U5 small nuclear ribonucleoprotein 27 kDa protein |
| O75157 | TSC22 domain family protein 2 |
| Q99607 | ETS-related transcription factor Elf-4 |
| **Accession** | **Protein names** |
| Q5HY92 | Fidgetin |
| Q9NX57 | Ras-related protein Rab-20 |
| Q9H2D1 | Mitochondrial folate transporter/carrier |
| Q9HB71 | Calcyclin-binding protein |
| P52954 | Transcription factor LBX1 |
| Q5BVD1 | TPA-induced transmembrane protein |
| Q5T8R8 | Uncharacterized protein C9orf66 |
| Q07812 | Apoptosis regulator BAX |
| Q9H5V9 | UPF0428 protein CXorf56 |
| Q9BS16 | Centromere protein K |
| P30566 | Adenylosuccinate lyase |
| Q8TAB7 | Putative coiled-coil domain-containing protein 26 |
| Q5QGT7 | Receptor-transporting protein 2 |
| Q6ZRF7 | Putative zinc finger protein 818 |
| O76076 | WNT1-inducible-signaling pathway protein 2 |
| O60507 | Protein-tyrosine sulfotransferase 1 |
| Q8NG50 | RAD52 motif-containing protein 1 |
| Q9Y473 | Zinc finger protein 175 |
| O00584 | Ribonuclease T2 |
| O00165 | HCLS1-associated protein X-1 |
| Q9P2F5 | Storkhead-box protein 2 |
| P11686 | Pulmonary surfactant-associated protein C |
| O95249 | Golgi SNAP receptor complex member 1 |
| Q9BPZ2 | Spindlin-2B;Spindlin-2A |
| Q9H2L4 | Transmembrane protein 60 |
| Q9Y2V3 | Retinal homeobox protein Rx |
| Q86U37 | Uncharacterized protein encoded by LINC01551 |
| Q9BTE0 | N-acetyltransferase 9 |
| Q5IS68 | Glutamate decarboxylase 1 |
| Q9H972 | Uncharacterized protein C14orf93 |
| Q96AQ7 | Cell death activator CIDE-3 |
