# Supplementary material for: Proteomic analysis of secreted proteins derived from amniotic fluid stem cells
Source: Cell Tissue Res. 2025 Jun 7;401(3):275–86. doi: 10.1007/s00441-025-03984-0 (PMC12411586; doi:10.1007/s00441-025-03984-0)
Supplement: Supplementary file 4 — Supplementary file4 (DOCX 44 KB) [file 441_2025_3984_MOESM4_ESM.docx]

**Table S3. List of 406 neuro-associated proteins in AFSC-se**

| **Accession** | **Protein names** |
| --- | --- |
| Q9NS98 | Semaphorin-3G |
| O14508 | Suppressor of cytokine signaling 2 |
| P25325 | 3-mercaptopyruvate sulfurtransferase |
| P01258 | Calcitonin;Calcitonin;Katacalcin |
| Q9HBG4 | V-type proton ATPase 116 kDa subunit a isoform 4 |
| P62068 | Ubiquitin carboxyl-terminal hydrolase 46 |
| Q9NP72 | Ras-related protein Rab-18 |
| P62851 | 40S ribosomal protein S25 |
| Q5VXT5 | Synaptophysin-like protein 2 |
| Q9UN67 | Protocadherin beta-10 |
| Q13449 | Limbic system-associated membrane protein |
| Q9UJZ1 | Stomatin-like protein 2, mitochondrial |
| P05388 | 60S acidic ribosomal protein P0 |
| Q6ZNC8 | Lysophospholipid acyltransferase 1 |
| Q07666 | KH domain-containing, RNA-binding, signal transduction-associated protein 1 |
| Q9UQN3 | Charged multivesicular body protein 2b |
| P55089 | Urocortin |
| Q9UBN1 | Voltage-dependent calcium channel gamma-4 subunit |
| Q7Z7J9 | Calcium/calmodulin-dependent protein kinase II inhibitor 1 |
| O15551 | Claudin-3 |
| P78415 | Iroquois-class homeodomain protein IRX-3 |
| P78334 | Gamma-aminobutyric acid receptor subunit epsilon |
| P15923 | Transcription factor E2-alpha |
| Q8N4L1 | Transmembrane protein 151A |
| P62841 | 40S ribosomal protein S15 |
| P48065 | Sodium- and chloride-dependent betaine transporter |
| P32322 | Pyrroline-5-carboxylate reductase 1, mitochondrial |
| Q12983 | BCL2/adenovirus E1B 19 kDa protein-interacting protein 3 |
| O15440 | Multidrug resistance-associated protein 5 |
| Q92581 | Sodium/hydrogen exchanger 6 |
| Q07817 | Bcl-2-like protein 1 |
| Q07001 | Acetylcholine receptor subunit delta |
| Q9Y5K8 | V-type proton ATPase subunit D |
| Q9P291 | Armadillo repeat-containing X-linked protein 1 |
| P51674 | Neuronal membrane glycoprotein M6-a |
| P07686 | Beta-hexosaminidase subunit beta;Beta-hexosaminidase subunit beta chain B;Beta-hexosaminidase subunit beta chain A |
| Q8WYJ6 | Septin-1 |
| Q9ULW3 | Activator of basal transcription 1 |
| O14544 | Suppressor of cytokine signaling 6 |
| P60484 | Phosphatidylinositol 3,4,5-trisphosphate 3-phosphatase and dual-specificity protein phosphatase PTEN |
| Q9Y5H0 | Protocadherin gamma-A3 |
| Q9Y320 | Thioredoxin-related transmembrane protein 2 |
| O75506 | Heat shock factor-binding protein 1 |
| Q8NA47 | Coiled-coil domain-containing protein 63 |
| **Accession** | **Protein names** |
| O75787 | Renin receptor |
| Q6ZSG2 | Protein FAM196A |
| Q07812 | Apoptosis regulator BAX |
| Q9NS82 | Asc-type amino acid transporter 1 |
| Q8NDC4 | MORN repeat-containing protein 4 |
| Q05066 | Sex-determining region Y protein |
| Q8NEE6 | F-box/LRR-repeat protein 13 |
| P03950 | Angiogenin |
| Q15365 | Poly(rC)-binding protein 1 |
| P57087 | Junctional adhesion molecule B |
| P06746 | DNA polymerase beta |
| P22466 | Galanin peptides;Galanin;Galanin message-associated peptide |
| P0C2W1 | F-box/SPRY domain-containing protein 1 |
| P60033 | CD81 antigen |
| O15245 | Solute carrier family 22 member 1 |
| Q9GZX9 | Twisted gastrulation protein homolog 1 |
| P61020 | Ras-related protein Rab-5B |
| Q9HCI5 | Melanoma-associated antigen E1 |
| O43665 | Regulator of G-protein signaling 10 |
| P26378 | ELAV-like protein 4;ELAV-like protein 2 |
| P09683 | Secretin |
| Q96A49 | Synapse-associated protein 1 |
| P52954 | Transcription factor LBX1 |
| Q9P2W1 | Homologous-pairing protein 2 homolog |
| P08908 | 5-hydroxytryptamine receptor 1A |
| P15514 | Amphiregulin |
| O95947 | T-box transcription factor TBX6 |
| P49427 | Ubiquitin-conjugating enzyme E2 R1 |
| P32248 | C-C chemokine receptor type 7 |
| Q5VWX1 | KH domain-containing, RNA-binding, signal transduction-associated protein 2 |
| Q92886 | Neurogenin-1 |
| Q14542 | Equilibrative nucleoside transporter 2 |
| O43761 | Synaptogyrin-3 |
| O95249 | Golgi SNAP receptor complex member 1 |
| O15498 | Synaptobrevin homolog YKT6 |
| O75840 | Krueppel-like factor 7 |
| O43868 | Sodium/nucleoside cotransporter 2 |
| P62937 | Peptidyl-prolyl cis-trans isomerase A;Peptidyl-prolyl cis-trans isomerase A, N-terminally processed;Peptidyl-prolyl cis-trans isomerase A-like 4A/B/C |
| Q5TA89 | Transcription factor HES-5 |
| P06730 | Eukaryotic translation initiation factor 4E |
| Q86YT5 | Solute carrier family 13 member 5 |
| Q9ULP9 | TBC1 domain family member 24 |
| O00501 | Claudin-5 |
| Q7Z5Q1 | Cytoplasmic polyadenylation element-binding protein 2 |
| P78543 | Protein BTG2 |
| Q9BXH1 | Bcl-2-binding component 3 |
| **Accession** | **Protein names** |
| Q14184 | Double C2-like domain-containing protein beta |
| P31146 | Coronin-1A |
| Q9NP59 | Solute carrier family 40 member 1 |
| Q12829 | Ras-related protein Rab-40B |
| Q9BUD6 | Spondin-2 |
| P02511 | Alpha-crystallin B chain |
| P36955 | Pigment epithelium-derived factor |
| Q9UK59 | Lariat debranching enzyme |
| Q99748 | Neurturin |
| Q99463 | Putative neuropeptide Y receptor type 6 |
| Q9UN72 | Protocadherin alpha-7 |
| O15049 | NEDD4-binding protein 3 |
| Q92536 | Y+L amino acid transporter 2 |
| Q99928 | Gamma-aminobutyric acid receptor subunit gamma-3 |
| Q86UD5 | Mitochondrial sodium/hydrogen exchanger 9B2 |
| P84095 | Rho-related GTP-binding protein RhoG |
| P10147 | C-C motif chemokine 3;MIP-1-alpha(4-69) |
| Q9P2A4 | ABI gene family member 3 |
| Q99578 | GTP-binding protein Rit2 |
| P11161 | E3 SUMO-protein ligase EGR2;Early growth response protein 1 |
| P30990 | Neurotensin/neuromedin N;Large neuromedin N;Neuromedin N;Neurotensin;Tail peptide |
| Q86WA9 | Sodium-independent sulfate anion transporter |
| Q15717 | ELAV-like protein 1 |
| P51452 | Dual specificity protein phosphatase 3 |
| P63000 | Ras-related C3 botulinum toxin substrate 1;Ras-related C3 botulinum toxin substrate 2 |
| Q16445 | Gamma-aminobutyric acid receptor subunit alpha-6 |
| Q8TAS1 | Serine/threonine-protein kinase Kist |
| Q96IG2 | F-box/LRR-repeat protein 20 |
| Q5IS62 | Neuropeptide Y receptor type 2 |
| Q9HBE1 | POZ-, AT hook-, and zinc finger-containing protein 1 |
| O43602 | Neuronal migration protein doublecortin |
| P47872 | Secretin receptor |
| Q13424 | Alpha-1-syntrophin |
| P22003 | Bone morphogenetic protein 5 |
| P08134 | Rho-related GTP-binding protein RhoC |
| P01116 | GTPase KRas;GTPase KRas, N-terminally processed |
| Q14549 | Homeobox protein GBX-1 |
| P62888 | 60S ribosomal protein L30 |
| P05090 | Apolipoprotein D |
| Q9NYK6 | Protein EURL homolog |
| Q99687 | Homeobox protein Meis3 |
| P32249 | G-protein coupled receptor 183 |
| Q8N6F1 | Claudin-19 |
| O15547 | P2X purinoceptor 6 |
| O60701 | UDP-glucose 6-dehydrogenase |
| **Accession** | **Protein names** |
| Q96B36 | Proline-rich AKT1 substrate 1 |
| Q04917 | 14-3-3 protein eta |
| P43686 | 26S protease regulatory subunit 6B |
| P46782 | 40S ribosomal protein S5;40S ribosomal protein S5, N-terminally processed |
| P46781 | 40S ribosomal protein S9 |
| P47898 | 5-hydroxytryptamine receptor 5A |
| P49207 | 60S ribosomal protein L34 |
| P39687 | Acidic leucine-rich nuclear phosphoprotein 32 family member A |
| O15143 | Actin-related protein 2/3 complex subunit 1B |
| O15144 | Actin-related protein 2/3 complex subunit 2 |
| O15511 | Actin-related protein 2/3 complex subunit 5 |
| Q7LC44 | Activity-regulated cytoskeleton-associated protein |
| Q15109 | Advanced glycosylation end product-specific receptor |
| P35368 | Alpha-1B adrenergic receptor |
| Q92186 | Alpha-2,8-sialyltransferase 8B |
| P53680 | AP-2 complex subunit sigma |
| O00189 | AP-4 complex subunit mu-1 |
| P53365 | Arfaptin-2 |
| Q5T4W7 | Artemin |
| P00505 | Aspartate aminotransferase, mitochondrial |
| P25106 | Atypical chemokine receptor 3 |
| P20273 | B-cell receptor CD22 |
| Q5SZJ8 | BEN domain-containing protein 6 |
| P01138 | Beta-nerve growth factor |
| Q6QNY1 | Biogenesis of lysosome-related organelles complex 1 subunit 2 |
| Q8TDH9 | Biogenesis of lysosome-related organelles complex 1 subunit 5 |
| Q9BSF8 | BTB/POZ domain-containing protein 10 |
| Q6ZWB6 | BTB/POZ domain-containing protein KCTD8 |
| P55287 | Cadherin-11 |
| Q96LZ3 | Calcineurin subunit B type 2 |
| P30988 | Calcitonin receptor |
| Q9HA72 | Calcium homeostasis modulator protein 2 |
| Q9Y691 | Calcium-activated potassium channel subunit beta-2 |
| P57796 | Calcium-binding protein 4 |
| Q8NCB2 | CaM kinase-like vesicle-associated protein |
| Q07343 | cAMP-specific 3,5-cyclic phosphodiesterase 4B |
| Q9GZU7 | Carboxy-terminal domain RNA polymerase II polypeptide A small phosphatase 1 |
| Q8NEV1 | Casein kinase II subunit alpha 3 |
| Q5IS54 | Caspase-3;Caspase-3 subunit p17;Caspase-3 subunit p12 |
| Q92583 | C-C motif chemokine 17 |
| Q9NTU7 | Cerebellin-4 |
| P09496 | Clathrin light chain A |
| Q8WVH0 | Complexin-3 |
| Q13324 | Corticotropin-releasing factor receptor 2 |
| Q86UF2 | cTAGE family member 6 |
| P56545 | C-terminal-binding protein 2 |
| **Accession** | **Protein names** |
| Q00535 | Cyclin-dependent-like kinase 5 |
| P20813 | Cytochrome P450 2B6 |
| P60026 | D(2) dopamine receptor |
| P21917 | D(4) dopamine receptor |
| Q08495 | Dematin |
| O94907 | Dickkopf-related protein 1 |
| Q9UBP4 | Dickkopf-related protein 3 |
| Q155Q3 | Dixin |
| Q969P6 | DNA topoisomerase I, mitochondrial |
| P09172 | Dopamine beta-hydroxylase;Soluble dopamine beta-hydroxylase |
| O60216 | Double-strand-break repair protein rad21 homolog |
| Q9UNE7 | E3 ubiquitin-protein ligase CHIP |
| Q99962 | Endophilin-A1 |
| P23769 | Endothelial transcription factor GATA-2 |
| P24530 | Endothelin B receptor |
| Q14511 | Enhancer of filamentation 1;Enhancer of filamentation 1 p55 |
| O43921 | Ephrin-A2 |
| P19419 | ETS domain-containing protein Elk-1 |
| O00303 | Eukaryotic translation initiation factor 3 subunit F |
| Q99502 | Eyes absent homolog 1 |
| Q5TGI0 | Failed axon connections homolog |
| Q9ULV1 | Frizzled-4 |
| Q13467 | Frizzled-5 |
| P24385 | G1/S-specific cyclin-D1 |
| O95452 | Gap junction beta-6 protein |
| P07492 | Gastrin-releasing peptide;Neuromedin-C |
| Q5IS68 | Glutamate decarboxylase 1 |
| Q9H4Y5 | Glutathione S-transferase omega-2 |
| P49841 | Glycogen synthase kinase-3 beta;Glycogen synthase kinase-3 alpha |
| Q8TE85 | Grainyhead-like protein 3 homolog |
| P12544 | Granzyme A |
| P10144 | Granzyme B |
| O15496 | Group 10 secretory phospholipase A2 |
| Q7Z4P5 | Growth/differentiation factor 7 |
| Q9H4S2 | GS homeobox 1 |
| P04899 | Guanine nucleotide-binding protein G(i) subunit alpha-2 |
| P50151 | Guanine nucleotide-binding protein G(I)/G(S)/G(O) subunit gamma-10 |
| P61952 | Guanine nucleotide-binding protein G(I)/G(S)/G(O) subunit gamma-11 |
| Q9UBI6 | Guanine nucleotide-binding protein G(I)/G(S)/G(O) subunit gamma-12 |
| P59768 | Guanine nucleotide-binding protein G(I)/G(S)/G(O) subunit gamma-2 |
| Q9UK08 | Guanine nucleotide-binding protein G(I)/G(S)/G(O) subunit gamma-8 |
| P62873 | Guanine nucleotide-binding protein G(I)/G(S)/G(T) subunit beta-1 |
| P62879 | Guanine nucleotide-binding protein G(I)/G(S)/G(T) subunit beta-2 |
| P16520 | Guanine nucleotide-binding protein G(I)/G(S)/G(T) subunit beta-3 |
| P11488 | Guanine nucleotide-binding protein G(t) subunit alpha-1 |
| P19086 | Guanine nucleotide-binding protein G(z) subunit alpha |

| **Accession** | **Protein names** |
| --- | --- |
| P63244 | Guanine nucleotide-binding protein subunit beta-2-like 1;Guanine nucleotide-binding protein subunit beta-2-like 1, N-terminally processed |
| Q9HAV0 | Guanine nucleotide-binding protein subunit beta-4 |
| Q9UBP5 | Hairy/enhancer-of-split related with YRPW motif protein 2 |
| O60243 | Heparan-sulfate 6-O-sulfotransferase 1 |
| P09429 | High mobility group protein B1 |
| Q04743 | Homeobox protein EMX2 |
| P56915 | Homeobox protein goosecoid |
| P28360 | Homeobox protein MSX-1 |
| Q9NSB8 | Homer protein homolog 2 |
| Q15011 | Homocysteine-responsive endoplasmic reticulum-resident ubiquitin-like domain member 1 protein |
| P00492 | Hypoxanthine-guanine phosphoribosyltransferase |
| P29218 | Inositol monophosphatase 1 |
| O14732 | Inositol monophosphatase 2 |
| P01584 | Interleukin-1 beta |
| O60259 | Kallikrein-8 |
| Q9Y664 | Kaptin |
| Q8NCW0 | Kremen protein 2 |
| Q9ULH4 | Leucine-rich repeat and fibronectin type-III domain-containing protein 2 |
| Q6PJG9 | Leucine-rich repeat and fibronectin type-III domain-containing protein 4 |
| Q6UY18 | Leucine-rich repeat and immunoglobulin-like domain-containing nogo receptor-interacting protein 4 |
| Q6UXK5 | Leucine-rich repeat neuronal protein 1 |
| O43300 | Leucine-rich repeat transmembrane neuronal protein 2 |
| Q7Z7J7 | Lipoma HMGIC fusion partner-like 4 protein |
| Q8N2G4 | Ly6/PLAUR domain-containing protein 1 |
| P39900 | Macrophage metalloelastase |
| P21757 | Macrophage scavenger receptor types I and II |
| P50281 | Matrix metalloproteinase-14 |
| P35240 | Merlin |
| Q9BRJ9 | Mesoderm posterior protein 1 |
| O15303 | Metabotropic glutamate receptor 6 |
| O00222 | Metabotropic glutamate receptor 8 |
| Q6P4Q7 | Metal transporter CNNM4 |
| P51608 | Methyl-CpG-binding protein 2 |
| Q9BQA1 | Methylosome protein 50 |
| Q9H492 | Microtubule-associated proteins 1A/1B light chain 3A |
| O96008 | Mitochondrial import receptor subunit TOM40 homolog |
| O43318 | Mitogen-activated protein kinase kinase kinase 7 |
| Q9BV23 | Monoacylglycerol lipase ABHD6 |
| P36021 | Monocarboxylate transporter 8 |
| Q15546 | Monocyte to macrophage differentiation factor |
| Q13485 | Mothers against decapentaplegic homolog 4 |
| P30307 | M-phase inducer phosphatase 3 |
| Q6DN12 | Multiple C2 and transmembrane domain-containing protein 2 |
| O00499 | Myc box-dependent-interacting protein 1 |
| **Accession** | **Protein names** |
| P58546 | Myotrophin |
| P41227 | N-alpha-acetyltransferase 10 |
| O95185 | Netrin receptor UNC5C |
| Q96CW9 | Netrin-G2 |
| Q9UMX5 | Neudesin |
| Q496H8 | Neuritin-like protein |
| Q5IS76 | Neuronal acetylcholine receptor subunit alpha-6 |
| O95502 | Neuronal pentraxin receptor |
| Q9GZT8 | NIF3-like protein 1 |
| P41146 | Nociceptin receptor |
| P23515 | Oligodendrocyte-myelin glycoprotein |
| P10451 | Osteopontin |
| P78380 | Oxidized low-density lipoprotein receptor 1;Oxidized low-density lipoprotein receptor 1, soluble form |
| P01178 | Oxytocin-neurophysin 1;Oxytocin;Neurophysin 1;Vasopressin-neurophysin 2-copeptin;Arg-vasopressin;Neurophysin 2;Copeptin |
| P11086 | Phenylethanolamine N-methyltransferase |
| Q92569 | Phosphatidylinositol 3-kinase regulatory subunit gamma |
| Q00169 | Phosphatidylinositol transfer protein alpha isoform |
| Q8IV08 | Phospholipase D3 |
| O15305 | Phosphomannomutase 2 |
| Q8IUK5 | Plexin domain-containing protein 1 |
| P22459 | Potassium voltage-gated channel subfamily A member 4 |
| P0CG38 | POTE ankyrin domain family member I |
| Q03052 | POU domain, class 3, transcription factor 1 |
| O75626 | PR domain zinc finger protein 1 |
| P49768 | Presenilin-1;Presenilin-1 NTF subunit;Presenilin-1 CTF subunit;Presenilin-1 CTF12 |
| Q96IZ0 | PRKC apoptosis WT1 regulator protein |
| Q86SP6 | Probable G-protein coupled receptor 149 |
| Q5UAW9 | Probable G-protein coupled receptor 157 |
| P01303 | Pro-neuropeptide Y;Neuropeptide Y;C-flanking peptide of NPY |
| P25789 | Proteasome subunit alpha type-4 |
| P28066 | Proteasome subunit alpha type-5 |
| P49720 | Proteasome subunit beta type-3 |
| O15234 | Protein CASC3 |
| Q99497 | Protein deglycase DJ-1 |
| P17252 | Protein kinase C alpha type |
| P24723 | Protein kinase C eta type |
| P41743 | Protein kinase C iota type |
| Q86TP1 | Protein prune homolog |
| P33763 | Protein S100-A5 |
| Q9NX38 | Protein Simiate |
| Q15532 | Protein SSXT |
| O00744 | Protein Wnt-10b |
| P56704 | Protein Wnt-3a |
| Q9Y6F9 | Protein Wnt-6 |
| **Accession** | **Protein names** |
| Q9H1J5 | Protein Wnt-8a |
| Q93098 | Protein Wnt-8b |
| Q9Y5E7 | Protocadherin beta-2 |
| Q9Y5G7 | Protocadherin gamma-A6 |
| Q9Y5G2 | Protocadherin gamma-B2 |
| Q96NT5 | Proton-coupled folate transporter |
| Q6A1A2 | Putative 3-phosphoinositide-dependent protein kinase 2;3-phosphoinositide-dependent protein kinase 1 |
| Q9BYX7 | Putative beta-actin-like protein 3;Putative beta-actin-like protein 3, N-terminally processed |
| P0C7U1 | Putative inactive neutral ceramidase B |
| Q96TA0 | Putative protocadherin beta-18 |
| P50395 | Rab GDP dissociation inhibitor beta |
| P46060 | Ran GTPase-activating protein 1 |
| P62491 | Ras-related protein Rab-11A;Ras-related protein Rab-11B |
| Q9H0T7 | Ras-related protein Rab-17 |
| Q92930 | Ras-related protein Rab-8B |
| P41220 | Regulator of G-protein signaling 2 |
| P49758 | Regulator of G-protein signaling 6 |
| Q9NRY4 | Rho GTPase-activating protein 35 |
| Q15669 | Rho-related GTP-binding protein RhoH |
| O00442 | RNA 3-terminal phosphate cyclase |
| Q92997 | Segment polarity protein dishevelled homolog DVL-3 |
| Q15019 | Septin-2 |
| O15269 | Serine palmitoyltransferase 1 |
| P37023 | Serine/threonine-protein kinase receptor R3 |
| Q52WX2 | Serine/threonine-protein kinase SBK1 |
| Q15173 | Serine/threonine-protein phosphatase 2A 56 kDa regulatory subunit beta isoform |
| Q9Y6A9 | Signal peptidase complex subunit 1 |
| O43805 | Sjoegren syndrome nuclear autoantigen 1 |
| Q99835 | Smoothened homolog |
| Q9NP91 | Sodium- and chloride-dependent transporter XTRP3 |
| Q9NY72 | Sodium channel subunit beta-3 |
| Q8NFF2 | Sodium/potassium/calcium exchanger 4 |
| Q9NQZ2 | Something about silencing protein 10 |
| Q96L92 | Sorting nexin-27 |
| Q9H228 | Sphingosine 1-phosphate receptor 5 |
| Q9NRA0 | Sphingosine kinase 2 |
| P57052 | Splicing regulator RBM11 |
| Q8WXA9 | Splicing regulatory glutamine/lysine-rich protein 1 |
| H3BQB6 | Stathmin domain-containing protein 1 |
| Q93045 | Stathmin-2 |
| O43704 | Sulfotransferase family cytosolic 1B member 1 |
| Q12824 | SWI/SNF-related matrix-associated actin-dependent regulator of chromatin subfamily B member 1 |
| Q05940 | Synaptic vesicular amine transporter |
| **Accession** | **Protein names** |
| O95473 | Synaptogyrin-4 |
| Q9BQG1 | Synaptotagmin-3 |
| O75558 | Syntaxin-11 |
| Q86Y82 | Syntaxin-12 |
| P61266 | Syntaxin-1B |
| P32856 | Syntaxin-2 |
| Q13190 | Syntaxin-5 |
| O00560 | Syntenin-1 |
| O14907 | Tax1-binding protein 3 |
| Q9UMR3 | T-box transcription factor TBX20 |
| Q9H2G4 | Testis-specific Y-encoded-like protein 2 |
| P36897 | TGF-beta receptor type-1 |
| Q86YJ6 | Threonine synthase-like 2 |
| P04216 | Thy-1 membrane glycoprotein |
| P01222 | Thyrotropin subunit beta |
| P23771 | Trans-acting T-cell-specific transcription factor GATA-3 |
| P15884 | Transcription factor 4 |
| P05549 | Transcription factor AP-2-alpha |
| Q92481 | Transcription factor AP-2-beta |
| Q01094 | Transcription factor E2F1 |
| O95416 | Transcription factor SOX-14;Transcription factor SOX-2;Transcription factor SOX-1 |
| Q9BWW7 | Transcriptional repressor scratch 1 |
| Q96QT4 | Transient receptor potential cation channel subfamily M member 7 |
| Q8NER1 | Transient receptor potential cation channel subfamily V member 1 |
| P57727 | Transmembrane protease serine 3 |
| Q4V9L6 | Transmembrane protein 119 |
| O95807 | Transmembrane protein 50A |
| Q8WUA8 | Tsukushin |
| P51451 | Tyrosine-protein kinase Blk |
| P07947 | Tyrosine-protein kinase Yes |
| Q9UMX0 | Ubiquilin-1 |
| O14933 | Ubiquitin/ISG15-conjugating enzyme E2 L6 |
| P62987 | Ubiquitin-60S ribosomal protein L40;Ubiquitin;60S ribosomal protein L40 |
| P60604 | Ubiquitin-conjugating enzyme E2 G2 |
| O43915 | Vascular endothelial growth factor D |
| Q9UIW0 | Ventral anterior homeobox 2 |
| Q96AJ9 | Vesicle transport through interaction with t-SNAREs homolog 1A |
| Q9UEU0 | Vesicle transport through interaction with t-SNAREs homolog 1B |
| Q15836 | Vesicle-associated membrane protein 3 |
| Q9BV40 | Vesicle-associated membrane protein 8 |
| Q9P0L0 | Vesicle-associated membrane protein-associated protein A |
| Q9P2U7 | Vesicular glutamate transporter 1;Vesicular glutamate transporter 2 |
| P21796 | Voltage-dependent anion-selective channel protein 1 |
| O95670 | V-type proton ATPase subunit G 2;V-type proton ATPase subunit G 1 |
| Q9H0C1 | Zinc finger MYND domain-containing protein 12 |
| P47974 | Zinc finger protein 36, C3H1 type-like 2 |
| **Accession** | **Protein names** |
| Q15915 | Zinc finger protein ZIC 1 |
| O95409 | Zinc finger protein ZIC 2 |
